# Efficacy of Off-Label Therapy for Non-alcoholic Fatty Liver Disease in Improving Non-invasive and Invasive Biomarkers: A Systematic Review and Network Meta-Analysis of Randomized Controlled Trials

**DOI:** 10.3389/fmed.2022.793203

**Published:** 2022-02-25

**Authors:** Qian Luo, Ruojun Wei, Yuzi Cai, Qihan Zhao, Yuning Liu, Wei Jing Liu

**Affiliations:** ^1^Dongzhimen Hospital Affiliated to Beijing University of Chinese Medicine, Beijing University of Chinese Medicine, Beijing, China; ^2^Key Laboratory of Chinese Internal Medicine of Ministry of Education and Beijing, Beijing, China; ^3^Zhanjiang Key Laboratory of Prevention and Management of Chronic Kidney Disease, Guangdong Medical University, Zhanjiang, China

**Keywords:** NAFLD, diabetes mellitus, vitamin E, pioglitazone, GLP-1 receptor agonists, SGLT2 inhibitors

## Abstract

**Objective:**

To evaluate the effects of vitamin E, pioglitazone, sodium-glucose cotransporter-2 (SGLT2) inhibitors, and glucagon-like peptide-1 (GLP-1) receptor agonists in patients with non-alcoholic fatty liver disease (NAFLD).

**Design:**

A network meta-analysis.

**Data Sources:**

PubMed, Embase, Cochrane Library, and Web of Science databases from their inception until September 1, 2021.

**Eligibility Criteria for Selecting Studies:**

Randomized controlled trials (RCTs) comparing the effects of four different drugs in patients with NAFLD were included. All superiority, non-inferiority, phase II and III, non-blinded, single-blinded, and double-blinded trials were included. Interventions of interest included vitamin E (α-tocopherol and δ-tocotrienol), pioglitazone, three kinds of GLP-1 receptor agonists (liraglutide, semaglutide, and dulaglutide), four SGLT2 inhibitors (dapagliflozin, empagliflozin, ipragliflozin, and tofogliflozin), and comparisons of these different drugs, and placebos.

**Main Outcome Measures:**

The outcome measures included changes in non-invasive tests [alanine aminotransferase (ALT), aspartate aminotransferase (AST), gamma-glutamyl transferase (GGT), controlled attenuation parameter (CAP), enhanced liver fibrosis (ELF) score, liver fat content (LFC), and keratin-18 (K-18)] and invasive tests [fibrosis score and resolution of non-alcoholic steatohepatitis (NASH)].

**Results:**

Twenty-seven trials including 3,416 patients were eligible for inclusion in the study. Results refer to vitamin E, pioglitazone, GLP-1 receptor agonists, and SGLT2 inhibitors. First, placebos were used as a reference. δ-Tocotrienol was superior to placebo in decreasing the GGT level. Semaglutide, ipragliflozin, and pioglitazone induced a significantly higher decrease in the ALT level than a placebo. Semaglutide, pioglitazone, and dapagliflozin were superior to placebo in decreasing the AST level. Tofogliflozin and pioglitazone induced a significantly higher decrease in the K-18 level than a placebo. Liraglutide was superior to placebo in decreasing CAP. Liraglutide, pioglitazone, and vitamin E induced a significantly higher increase in resolution of NASH than a placebo. As for pairwise comparisons, semaglutide and pioglitazone were superior to liraglutide in decreasing the ALT level. Semaglutide induced a significantly higher decrease in the ALT level than dulaglutide. Semaglutide was obviously superior to empagliflozin, liraglutide, dulaglutide, and tofogliflozin in decreasing the AST level. Pioglitazone induced a significantly higher decrease in the GGT level than ipragliflozin. δ-Tocotrienol was superior to liraglutide in decreasing the GGT level. Tofogliflozin and pioglitazone induced a significantly higher decrease in the K-18 level than dulaglutide. Pioglitazone was superior to vitamin E in increasing the resolution of NASH. Furthermore, liraglutide treatment had the highest SUCRA ranking in decreasing CAP and ELF scores and increasing the resolution of NASH. Pioglitazone treatment had the highest SUCRA ranking in decreasing LFC and fibrosis scores. Tofogliflozin treatment had the highest SUCRA ranking in decreasing K-18, while dapagliflozin treatment had the highest SUCRA ranking in decreasing the GGT level. Semaglutide treatment had the highest SUCRA ranking in decreasing the levels of ALT and AST.

**Conclusion:**

The network meta-analysis provided evidence for the efficacy of vitamin E, pioglitazone, SGLT2 inhibitors, and GLP-1 receptor agonists in treating patients with NAFLD. To find the best guide-level drugs, it is necessary to include more RCTs with these off-label drugs, so that patients and clinicians can make optimal decisions together.

**Systematic Review Registration:**

https://www.crd.york.ac.uk/prospero, identifier: CRD42021283129.

## Introduction

Non-alcoholic fatty liver disease (NAFLD) is defined by the presence of steatosis in more than 5% of hepatocytes, in association with metabolic risk factors (in particular, obesity and type 2 diabetes) and in the absence of excessive alcohol consumption (≥30 g/day for men and ≥20 g/day for women) or other chronic liver diseases (1). According to epidemiological reports, NAFLD occurs in 47.3–63.7% of individuals with type 2 diabetes. As an established risk factor of NAFLD, diabetes contributes to the progression of non-alcoholic steatohepatitis (NASH) and the occurrence of hepatocellular carcinoma (HCC) (2–4). In patients with diabetes, NAFLD can accelerate the progression to cirrhosis, end-stage liver disease, and HCC (5). Therefore, it is extremely important to treat NAFLD with or without diabetes in a timely manner. At present, there are various unapproved drugs for NASH. However, it is still unclear whether these drugs can optimize the existing therapies [weight loss and exercise (6, 7)] to achieve improvement in two liver histological endpoints (NASH resolution without worsening of fibrosis; or an improvement in fibrosis of one stage or more without worsening of NASH) (8). Glucagon-like peptide-1 (GLP-1) receptor agonists and SGLT2 inhibitors are two kinds of common off-label hypoglycemic drugs. It has been shown that GLP-1 receptor agonists can control energy intake and body weight by lowering blood glucose or by prolonging gastric emptying and inducing satiety to reduce liver damage caused by any metabolic factor (9). SGLT2 inhibitors could reduce inflammatory markers, increase the oxidation of free fatty acids, and improve insulin resistance; they could also increase ketone body metabolism and improve fatty liver injury by upregulating ketogenic enzymes and transporters in the liver (10). In addition, vitamin E and pioglitazone have been endorsed by the current guidelines (Asia-Pacific Working Party on NAFLD) but not yet approved by the US Food and Drug Administration (FDA) and European Medicines Agency (EMA) guidelines in treating NAFLD linked with diabetes mellitus (recently renamed MAFLD) (11). Pioglitazone is a peroxisome proliferator-activated receptor γ (PPARγ) agonist, which can protect the liver by improving insulin resistance in patients with NAFLD and with type 2 diabetes mellitus. Although the mechanism is unclear, it has been shown that pioglitazone could improve biochemical and histological parameters (without diabetes) (12). In addition, one study had shown that the antioxidant effect of vitamin E (α-tocopherol) could significantly improve the disease progression of patients with NASH (13). GLP-1 receptor agonists and SGLT2 inhibitors are two kinds of hypoglycemic agents that are currently undergoing phase II and III trials. It has been shown that these drugs can protect the heart and kidneys of patients with NAFLD and with type 2 diabetes and induce weight loss (14–16). Several trials in patients with NAFLD have shown that pioglitazone, a drug from the class of thiazolidinediones, improve NASH activity and fibrosis (17). In addition, vitamin E has been found to be useful for patients with NAFLD and without type 2 diabetes (18).

The detection methods for NAFLD can be divided into non-invasive and invasive methods. The non-invasive methods lack specificity and sensitivity, and poorly correlate with histological severity [alanine aminotransferase (ALT), aspartate aminotransferase (AST), and gamma-glutamyl transferase (GGT)], whereas the invasive method of biopsy is the golden standard for NAFLD diagnosis, but it has a high operating threshold and is prone to sampling errors. The majority of several previous network meta-analyses (7, 19, 20) have analyzed liver enzyme concentrations and a wide range of scores rather than invasive procedures such as liver biopsy. Here, we conducted a network meta-analysis of randomized controlled trials (RCTs) with the aim to directly and indirectly compare the effects of vitamin E, pioglitazone, GLP-1 receptor agonists, SGLT2 inhibitors, and a placebo for patients with NAFLD, thereby providing new potential clinical drugs for NAFLD. In addition to reporting the routine indicators as in the previous NMAs, we also added several non-invasive NAFLD detection methods closely related to the progression of liver histology, such as controlled attenuation parameter (CAP) and enhanced liver fibrosis (ELF) score. CAP (11) could be used to evaluate liver steatosis, while ELF score has been recognized by the United Kingdom's National Institute for Health and Clinical Excellence (NICE) and other associations in the UK for the evaluation and monitoring of patients suspected of NAFLD among metabolic risk factors (21).

## Methods

### Data Sources and Literature Search

We performed an extensive search of the PubMed, Embase, Cochrane Library, and Web of Science databases from their inception until September 1, 2021, for RCTs investigating different agents for treating patients with NAFLD. Additional studies were searched in the reference lists of all identified publications, including relevant meta-analyses. Search strategies are shown in Supplementary Material.

### Study Selection

First, two reviewers (Luo and Cai) independently screened records according to the title/abstract and then screened the full text of the relevant records against the predefined selection criteria. Namely, eligible papers were RCTs that compared the effects of different treatments of patients with NAFLD. All superiority, non-inferiority, phase II and III, non-blinded, single-blinded, and double-blinded trials were included. Interventions of interest included vitamin E (α-tocopherol and δ-tocotrienol), pioglitazone, three kinds of GLP-1 receptor agonists (liraglutide, semaglutide, and dulaglutide), four types of SGLT2 inhibitors (dapagliflozin, empagliflozin, ipragliflozin, and tofogliflozin), and comparisons of these different drugs, and placebos. The outcome measures included changes in non-invasive tests [ALT, AST, GGT, CAP, liver fat content (LFC) on MRI–PDFF or H-MRS, ELF score, keratin-18 (K-18) M30 fragment] and invasive tests (fibrosis score and resolution of NASH). At the same time, we excluded clinical studies with the following features: (1) the presence of other conditions that may not be associated with NAFLD as identified by history, physical examination, and laboratory investigations; (2) the examination of a single drug for treating patients with NAFLD (e.g., comparison of different doses or frequencies of the same drug); and (3) the data of outcome indicators being unavailable, even after contacting the authors.

### Data Extraction and Risk-of-Bias Assessment

Two reviewers (Luo and Wei) independently extracted data from the original trial reports using a standardized form (including author, sample size, sex, age, and outcome index), and then double-checked the extracted data. We assessed the risk of bias of the individual studies using the Cochrane Risk of Bias Tool (21), which is based on the following domains: random sequence generation, allocation concealment, blinding of participants and personnel, blinding of outcome assessment, incomplete outcome data, selective outcome reporting, and other sources of bias. Items were scored as low, high, or unclear risk of bias. Two investigators (Luo and Wei) independently conducted study selection and data extraction. Two investigators (Luo and Cai) independently assessed the risk of bias of the individual studies. Any discrepancies were resolved by consensus and arbitration by a panel of adjudicators (Luo, Cai, Zheng, Zhao, and Guo) (18, 19).

### Data Analyses

The statistical method for network meta-analysis was based on a frequency framework. All outcome measures were analyzed using a random-effects model for data analysis, except for two indicators (K-18 and CAP, for which we used the fixed-effects model). If the evaluated indicators were continuous variables, standardized mean difference (SMD) or mean difference (MD) was used for the determination of effect size; if they were binary variables, odds ratio (OR) was used as the effect size, and the corresponding 95% CI was calculated. Multiple independent groups in a study (e.g., different semaglutide doses) were considered separate datasets. More than one record of treatment duration in one study (23) was divided into independent datasets. Stata16.0 software (StataCorp, Texas, USA) was used to select the frequentist framework random-effects model for network meta-analysis; the ranking group command was used for data preprocessing; the network relationship diagram was drawn for the comparison between the interventions of each outcome indicator; the efficacy ranking was performed; and the cumulative probability ranking diagram was drawn to obtain the area under the curve (SUCRA). In the reticulation diagram, dot size represents the number of patients with relevant interventions, and the thickness of the line between points represents the number of included studies (22). SUCRA was expressed as a percentage. When SUCRA was 100%, it indicated that the intervention was absolutely effective, and when it was 0, it indicated that the intervention was absolutely ineffective. The inconsistency test was mainly used to assess the degree of consistency between the direct comparison results and the indirect comparison results. Finally, the presence of small sample effects in the network was identified by drawing a funnel plot (publication bias was only examined if ≥10 study comparisons were included in the analysis). Review Manager 5.4 software (Nordic Cochrane Centre) was used for literature quality evaluation in this study.

## Results

### Study Characteristics

**Figure 1:**
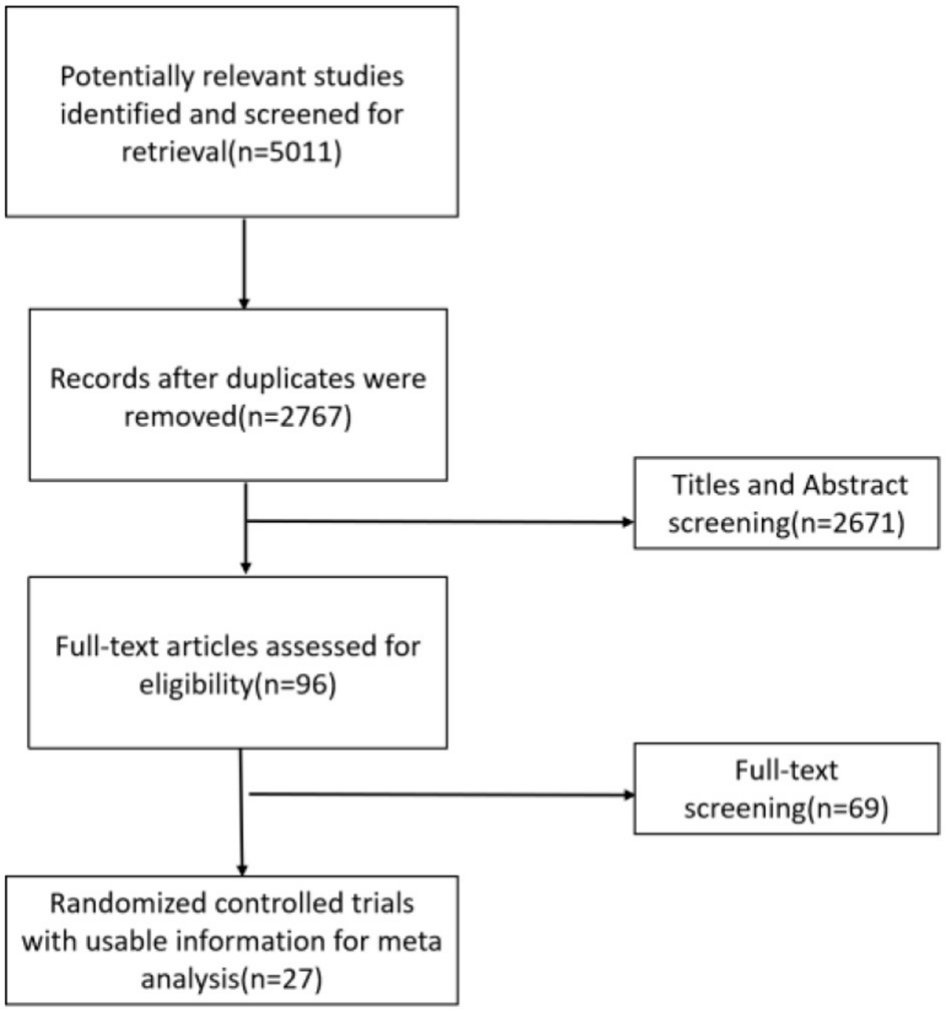
Flow chart of literature search and selection.

A total of 27 eligible studies, published between 1989 and 2021, corresponding to 3,416 adults, were selected for pooled analyses (7, 17–20, 23–44). Five (23, 29, 30, 32, 37) of them were ≥3-arm trials, and the remaining 22 were double-arm trials. The baseline characteristics of the included RCTs are provided in Supplementary Table 1. The literature search process is shown in Figure 1.

### Quality of the Included Studies

**Figure 2:**
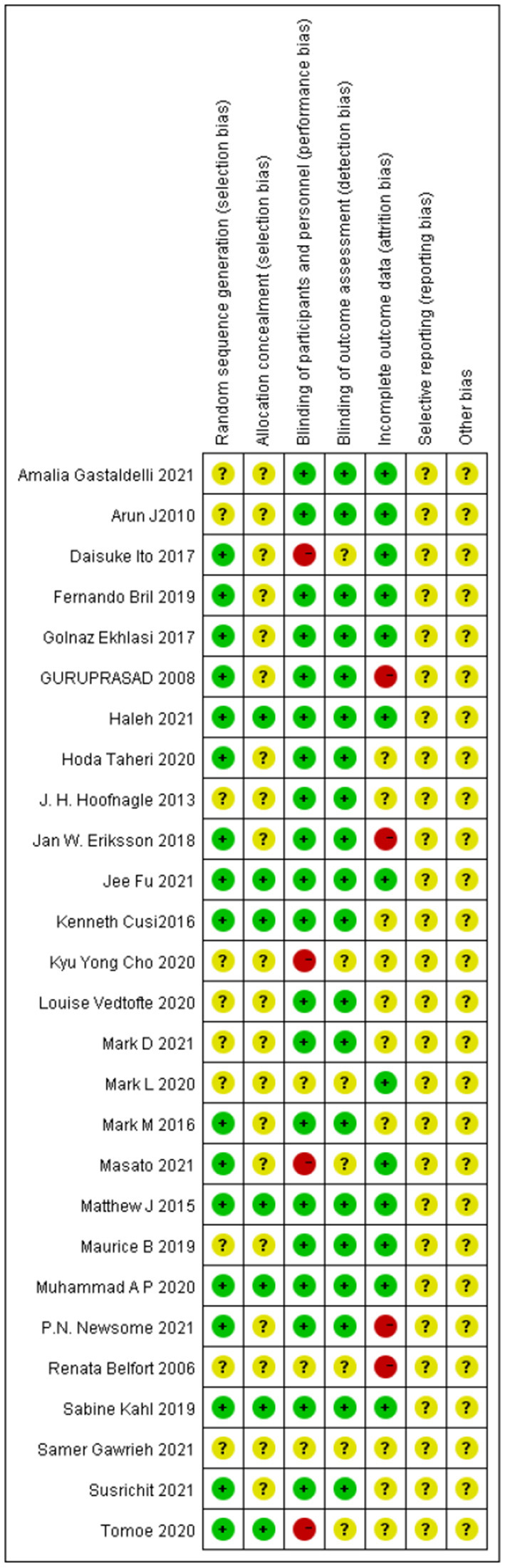
Risk-of-bias summary using the SYRCLE risk-of-bias tool.

**Figure 3:**
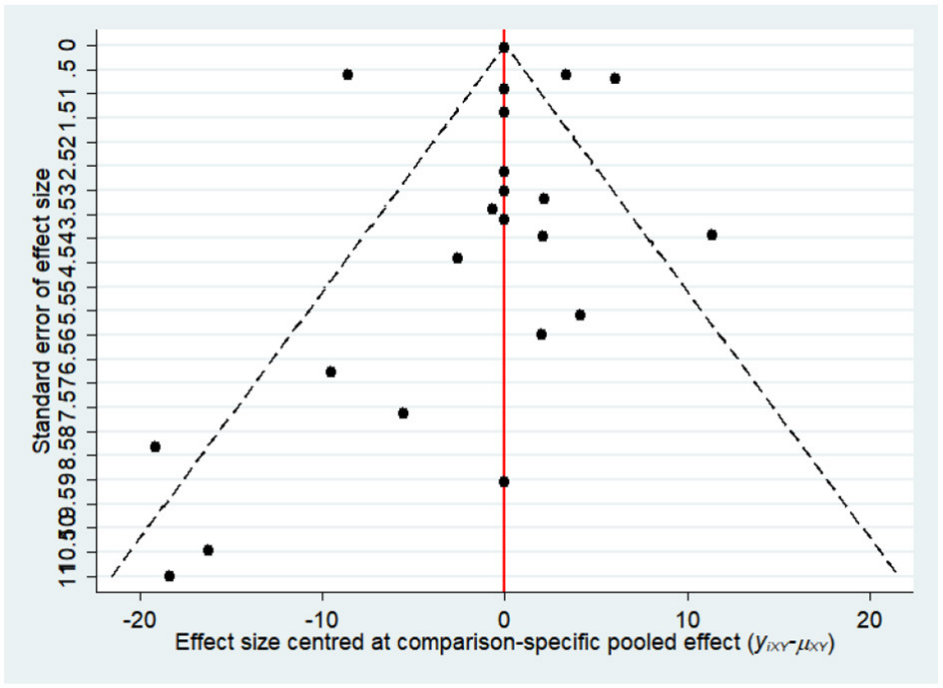
The funnel plot for alanine aminotransferase (ALT).

**Figure 4:**
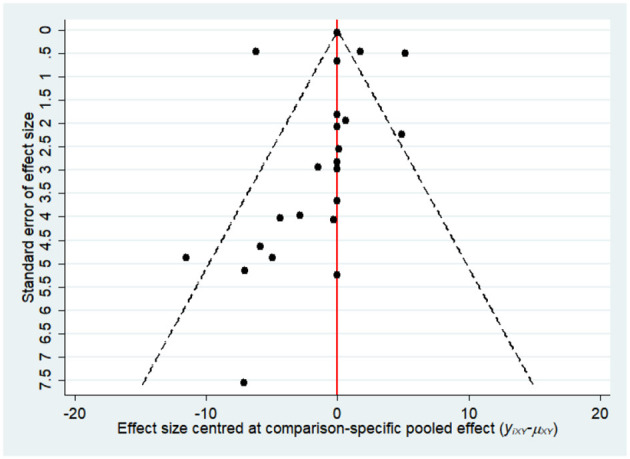
The funnel plot for aspartate aminotransferase (AST).

**Figure 5:**
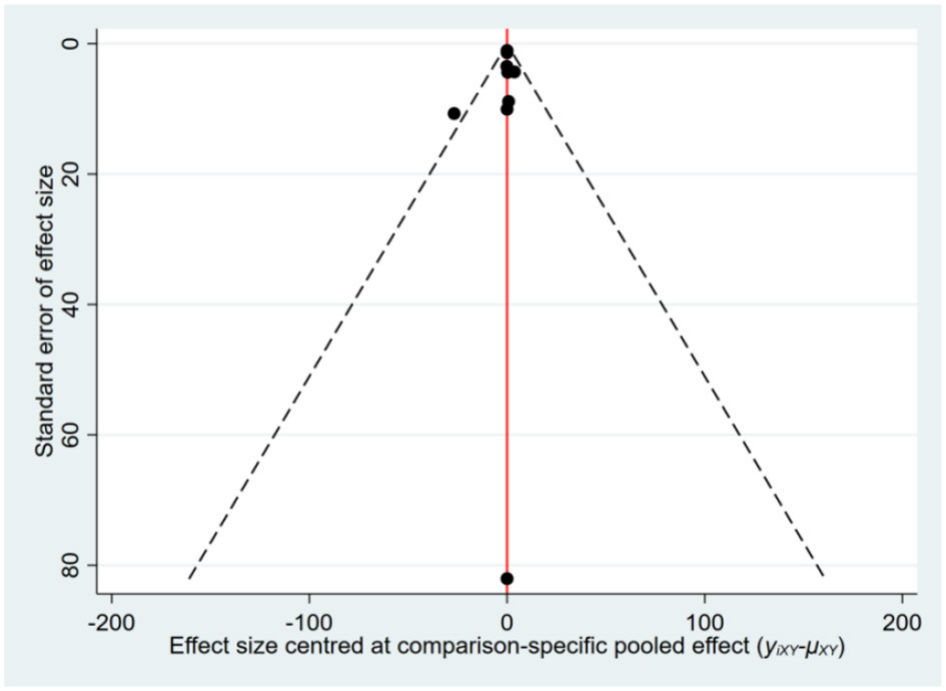
The funnel plot for gamma-glutamyl transferase (GGT).

Most of the studies were judged to be at low or unclear risk of bias for the six domains according to the Cochrane Collaboration's tool. Four studies (28, 33, 36, 44) were judged to be at a high risk of bias for blinding participants and personnel due to performing single-blinding or open-label trials. Four studies (17, 26, 30, 31) were judged to be at a high risk of bias for incomplete outcome data due to the high rate of dropout. The risk-of-bias assessment of the trials included in this study is presented in Figure 2. In addition, the funnel plot results suggested there may be some publication bias in ALT, AST, and GGT, as shown in Figures 3–5.

### Network Meta-Analysis Results

**Figure 6:**
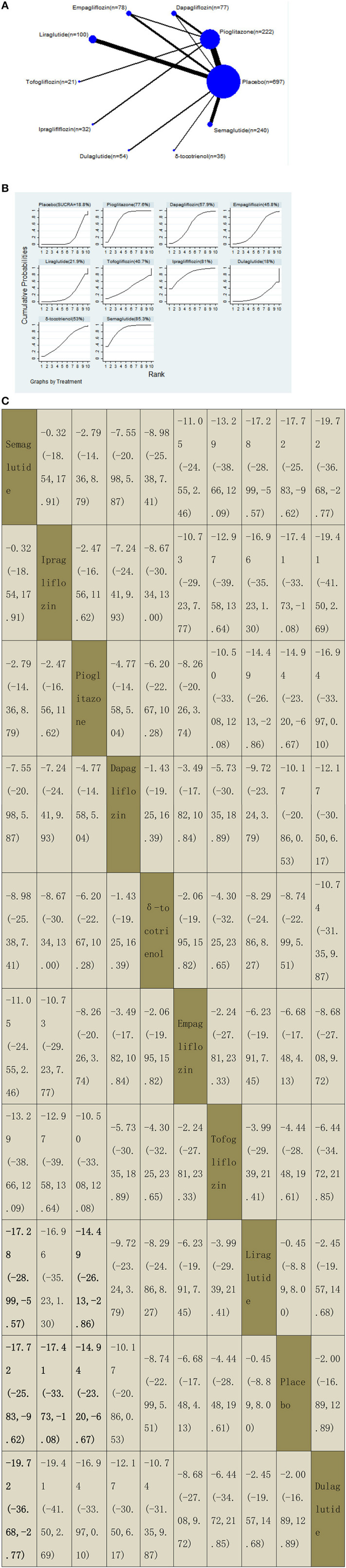
Network graphs **(A)**, pairwise comparisons **(B)**, and SUCRA probability ranking **(C)** for alanine aminotransferase (ALT).

**Figure 7:**
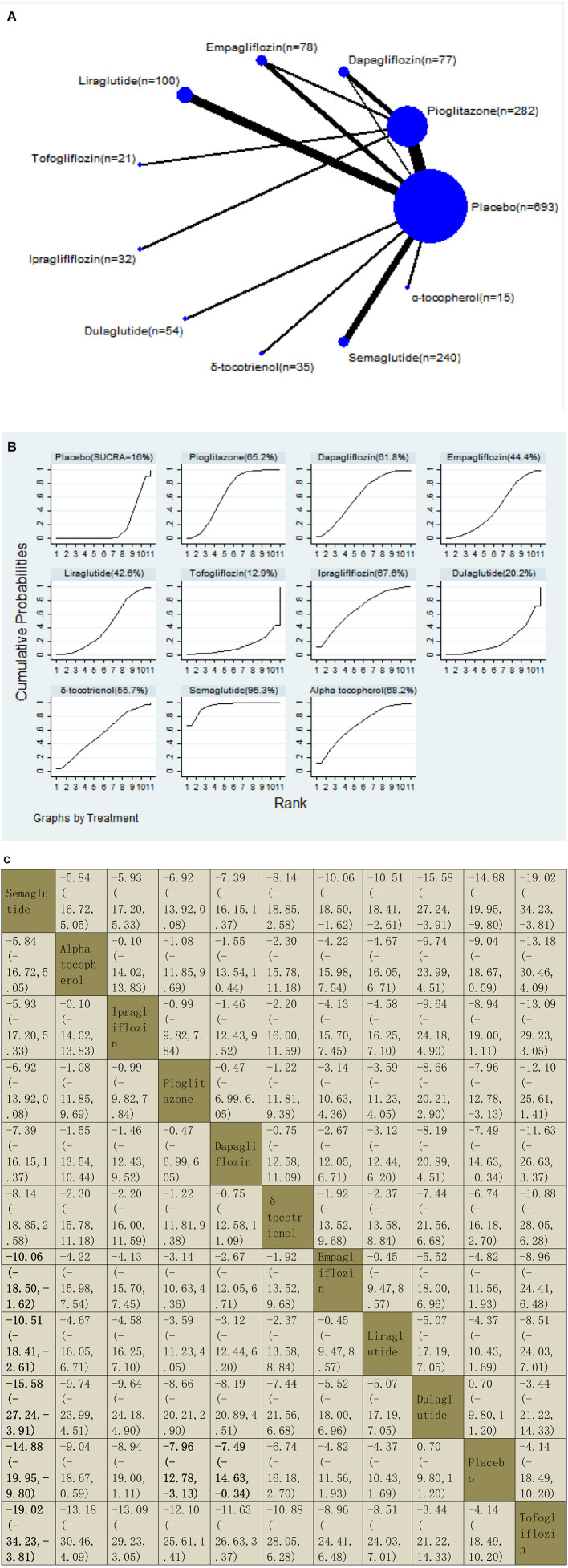
Network graphs **(A)**, pairwise comparisons **(B)**, and SUCRA probability ranking **(C)** for aspartate aminotransferase (AST).

**Figure 8:**
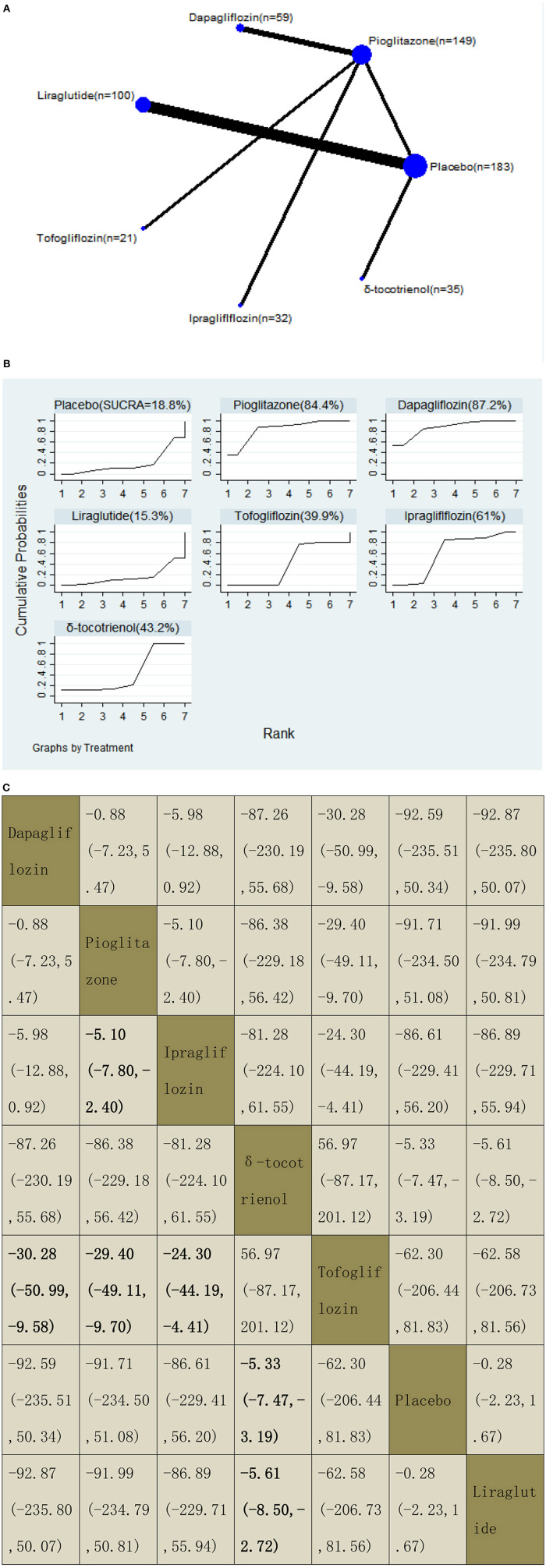
Network graphs **(A)**, pairwise comparisons **(B)**, and SUCRA probability ranking **(C)** for gamma-glutamyl transferase (GGT).

**Figure 9:**
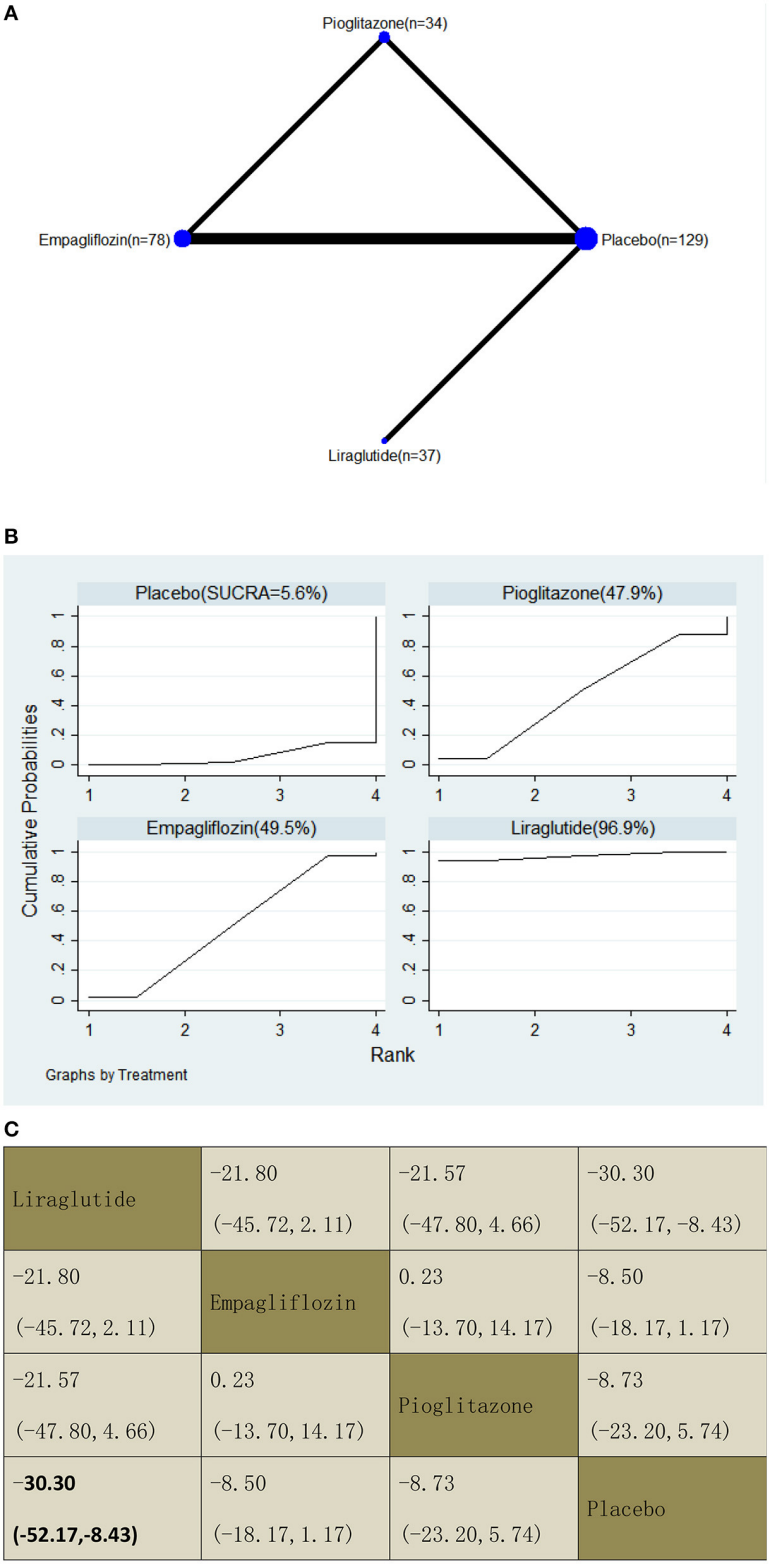
Network graphs **(A)**, pairwise comparisons **(B)**, and SUCRA probability ranking **(C)** for controlled attenuation parameter (CAP).

**Figure 10:**
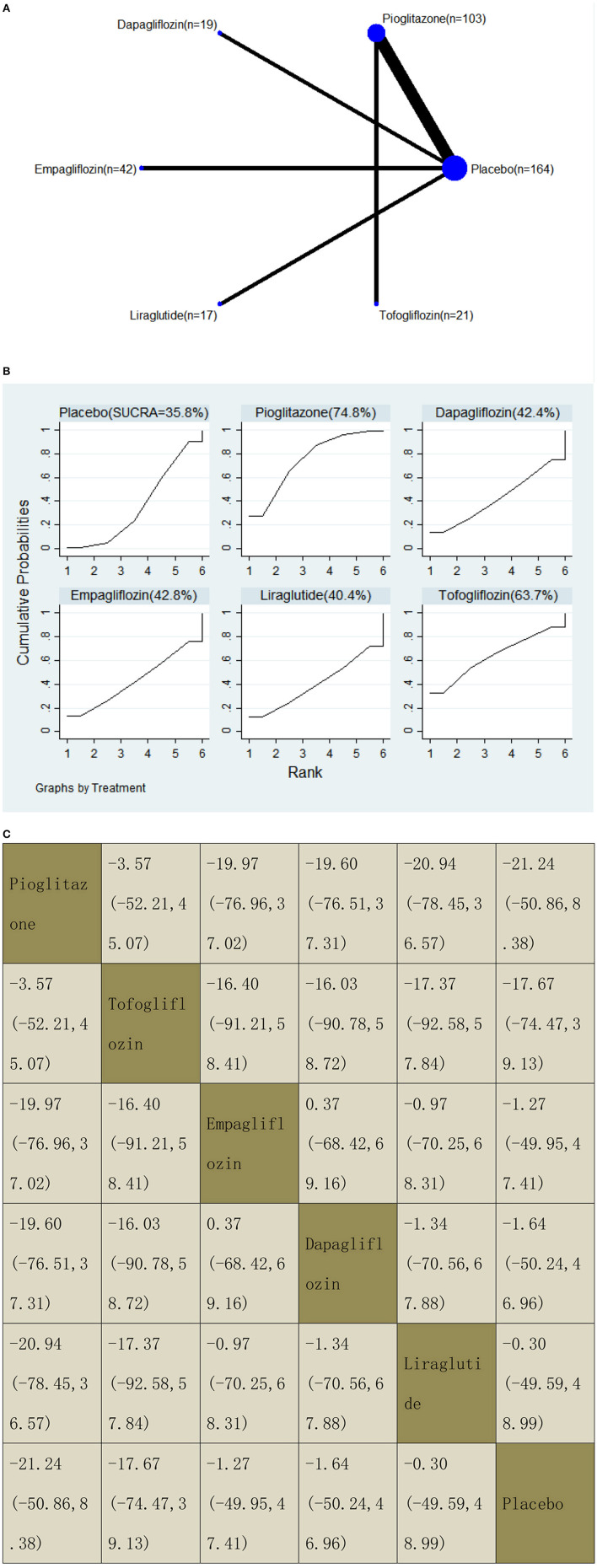
Network graphs **(A)**, pairwise comparisons **(B)**, and SUCRA probability ranking **(C)** for liver fat content (LFC).

**Figure 11:**
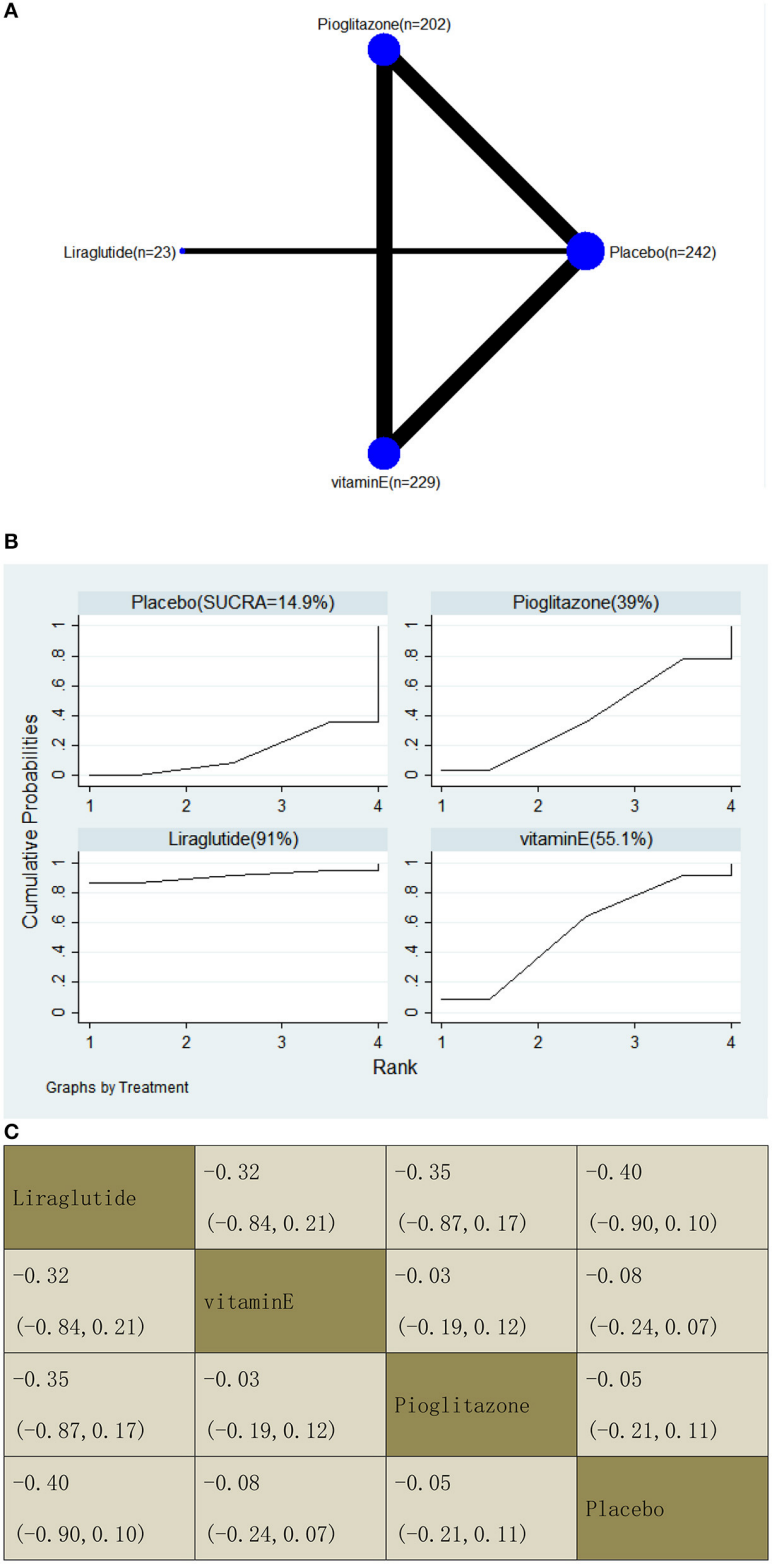
Network graphs **(A)**, pairwise comparisons **(B)**, and SUCRA probability ranking **(C)** for enhanced liver fibrosis (ELF) score.

**Figure 12:**
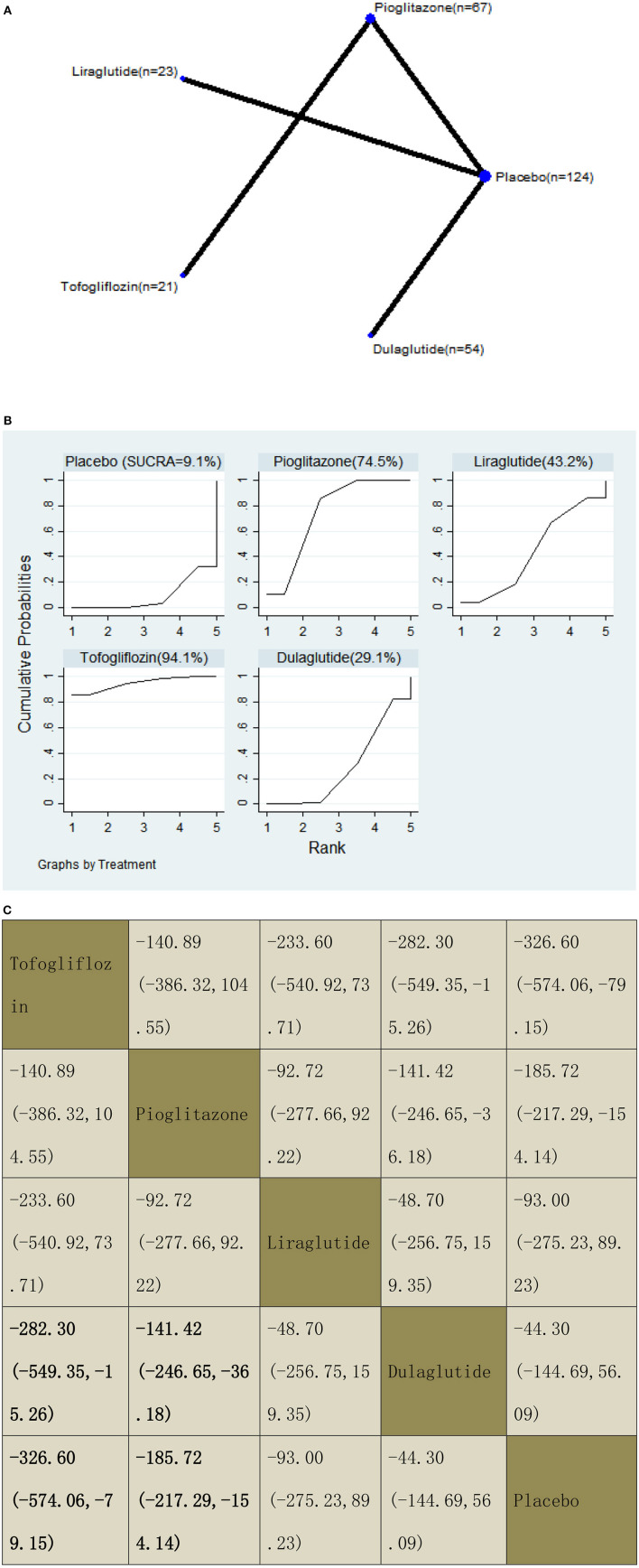
Network graphs **(A)**, pairwise comparisons **(B)**, and SUCRA probability ranking **(C)** for keratin-18 (K-18).

**Figure 13:**
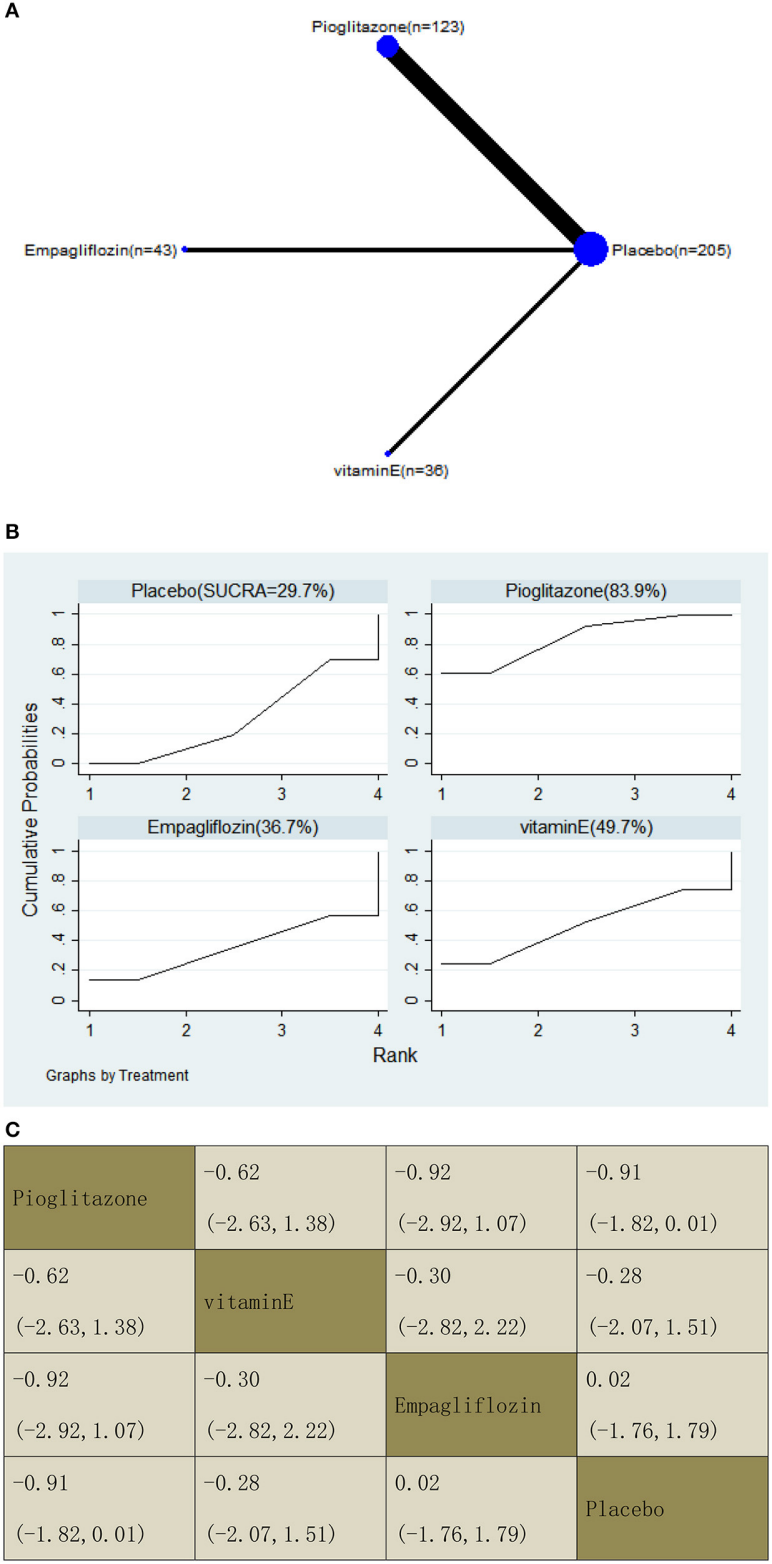
Network graphs **(A)**, pairwise comparisons **(B)**, and SUCRA probability ranking **(C)** for fibrosis score.

**Figure 14:**
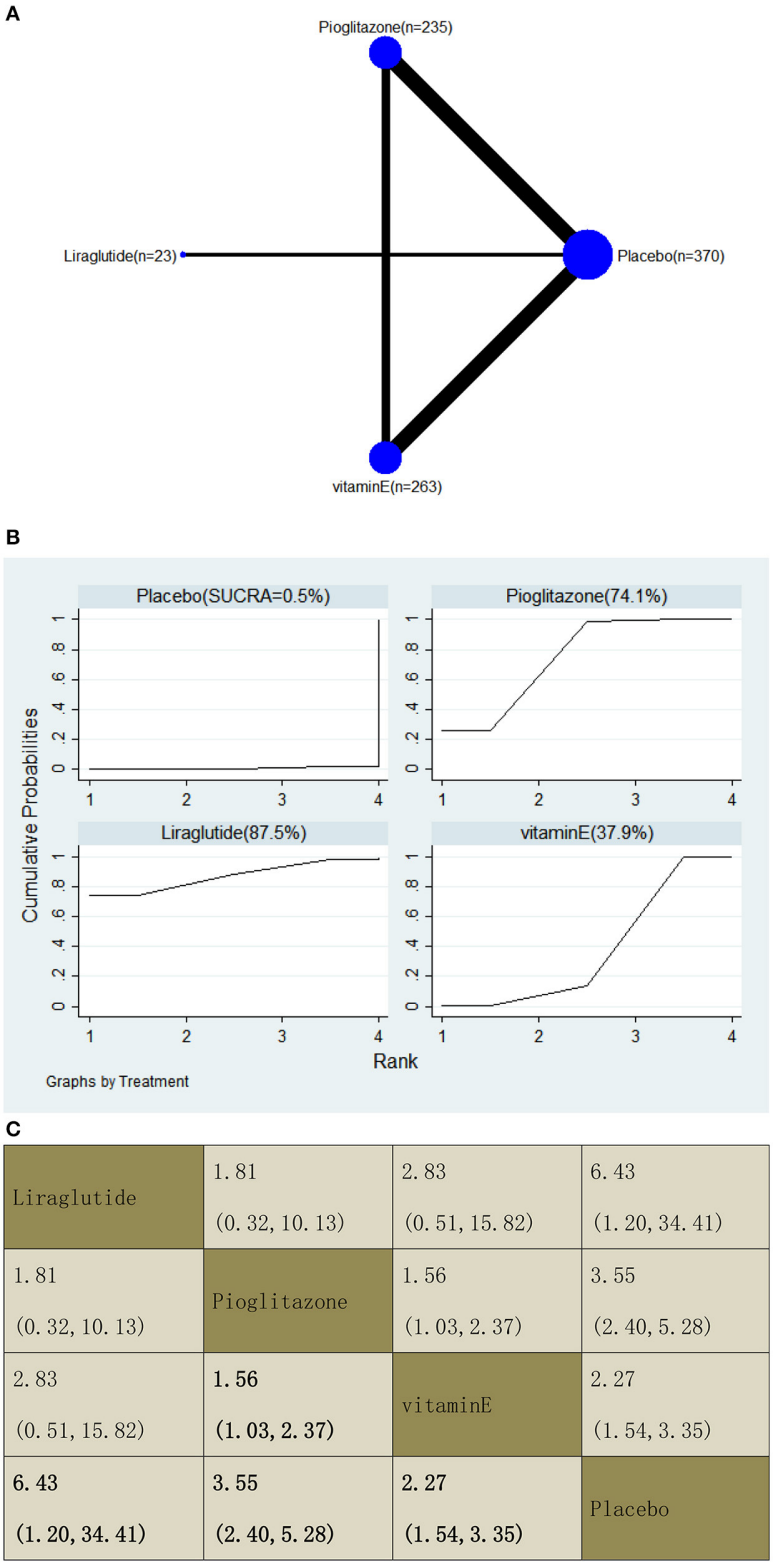
Network graphs **(A)**, pairwise comparisons **(B)**, and SUCRA probability ranking **(C)** for resolution of non-alcoholic steatohepatitis (NASH).

We assessed the efficacy of four different off-label agents, including three different GLP-1 receptor agonists, four kinds of SGLT2 inhibitors, vitamin E, and pioglitazone for patients with NAFLD. Figures 6A–14A show network graphs of all the drugs included in the study. The width of the lines is proportional to the number of trials comparing each pair of treatments. The sizes of the circles are proportional to the number of patients.

### Non-invasive Tests

#### Alanine Aminotransferase

Our NMA included 20 RCTs reporting the administration of vitamin E, pioglitazone, three GLP-1 receptor agonists, and four SGLT2 inhibitors among 1,506 patients. A placebo was used as a reference. We found that three drugs significantly decreased the ALT levels, with MDs of −17.72 (95% CI: −25.83, −9.62) for semaglutide, −17.41 (−33.73, −1.08) for ipragliflozin, and −14.94 (−23.20, −6.67) for pioglitazone. Compared with liraglutide, semaglutide and pioglitazone significantly decreased the ALT levels, with MDs of −17.28 (−28.99, −5.57) for semaglutide and −14.49 (−26.13, −2.86) for pioglitazone. In addition, semaglutide decreased the ALT levels compared with dulaglutide, with MDs of −19.72 (−36.68, −2.77). The results of pairwise comparisons are indicated by the MDs and 95% CI (Figure 6B). According to the results of SUCRA, semaglutide may be the most effective intervention to reduce the level of ALT in patients with NAFLD, and the results of the SUCRA probability ranking are shown in Figure 6C.

#### Aspartate Aminotransferase

Our NMA included 21 RCTs reporting the administration of vitamin E, pioglitazone, three GLP-1 receptor agonists, and four SGLT2 inhibitors among 1,597 patients. We found that semaglutide could significantly decrease the levels of AST than five drugs, with MDs of −10.06 (95% CI: −18.50, −1.62) than empagliflozin, −10.51 (−18.41, −2.61) than liraglutide, −15.58 (−27.24, −3.91) than dulaglutide, −14.88 (−19.95, −9.80) than placebo, and −19.02 (−34.23, −3.81) than tofogliflozin. Compared with a placebo, three included agents significantly decreased the levels of AST, with MDs of −14.88 (−19.95, −9.80) for semaglutide, −7.96 (−12.78, −3.13) for pioglitazone, and −7.49 (−14.63, −0.34) for dapagliflozin. The results of the pairwise comparison are indicated by the MDs and 95% CI (Figure 7B). According to the SUCRA results, semaglutide may be the most effective intervention to reduce the level of AST in patients with NAFLD, and the results of the SUCRA probability ranking are shown in Figure 7C.

#### Gamma-Glutamyl Transferase

Our NMA included 10 RCTs reporting the administration of vitamin E, pioglitazone, one GLP-1 receptor agonist, and three SGLT2 inhibitors among 579 patients. Tofogliflozin was used as a reference. We found that three drugs could significantly decrease the levels of GGT, with MDs of −30.28 (95% CI: −50.99, −9.58) for dapagliflozin, −29.40 (−49.11, −9.70) for pioglitazone, and −24.30 (−44.19, −4.41) for ipragliflozin. δ-Tocotrienol decreased the levels of GGT significantly compared with placebo and liraglutide, with MDs of −5.33 (−7.47, −3.19) compared with placebo and −5.61 (−8.50, −2.72) compared with liraglutide. We also found that pioglitazone decreased the levels of GGT significantly compared with ipragliflozin, with MDs of −5.10 (−7.80, −2.40). The results of the pairwise comparison are indicated by the MDs and 95% CI (Figure 8B). According to the SUCRA results, dapagliflozin may be the most effective intervention to reduce the level of GGT in patients with NAFLD, and the results of the SUCRA probability ranking are shown in Figure 8C.

#### Controlled Attenuation Parameter

With regard to CAP decrease, our NMA included three RCTs, reporting the administration of pioglitazone, one GLP-1 receptor agonist, and one SGLT2 inhibitor among 278 patients. Liraglutide decreased the CAP significantly compared with a placebo, with MDs of −30.30 (95% CI: −52.17, −8.43). The results of the pairwise comparison are indicated by the MDs and 95% CI (Figure 9B). According to the SUCRA results, liraglutide may be the most effective intervention to reduce the CAP of patients with NAFLD, and the results of the SUCRA probability ranking are shown in Figure 9C.

#### Liver Fat Content on MRI–PDFF or H-MRS

With regard to LFC decrease, our NMA included seven RCTs, reporting the administration of pioglitazone, one GLP-1 receptor agonist, and three SGLT2 inhibitors among 366 patients. There was no statistically significant difference between the five drugs and a placebo, i.e., for pioglitazone −21.24 (95% CI: −50.86, 8.38), tofogliflozin −17.67 (−74.47, 39.13), empagliflozin −1.27 (−49.95, 47.41), dapagliflozin −1.64 (−50.24, 46.96), and liraglutide −0.30 (−49.59, 48.99). The results of the pairwise comparison are indicated by the MDs and 95% CI (Figure 10B). According to the SUCRA results, pioglitazone may be the most effective intervention to reduce the LFC of patients, and the results of the SUCRA probability ranking are shown in Figure 10C.

#### Enhanced Liver Fibrosis Score

With regard to a decrease in the ELF score, our NMA included two RCTs, reporting the administration of vitamin E, pioglitazone, and one GLP-1 receptor agonist among 696 patients. There was no statistically significant difference between the three drugs and placebo, i.e., for liraglutide −0.40 (95% CI: −0.90, 0.10), vitamin E −0.08 (−0.24, 0.07), and pioglitazone −0.05 (−0.21, 0.11). The results of the pairwise comparison are indicated by the MDs and 95% CI (Figure 11B). According to the results of SUCRA, liraglutide may be the most effective intervention to reduce the ELF score of patients, and the results of the SUCRA probability ranking are shown in Figure 11C.

#### Keratin-18 (K-18) M30 Fragment

With regard to a decrease in K-18, our NMA included four RCTs, reporting the administration of pioglitazone, two GLP-1 receptor agonists, and one SGLT2 inhibitor among 289 patients. Compared with dulaglutide, two included agents significantly decreased the K-18 levels, with MDs of −282.30 (95% CI: −549.35, −15.26) for tofogliflozin and −141.42 (−246.65, −36.18) for pioglitazone. Also, compared with placebo, two included agents significantly decreased the K-18 levels, with MDs of −326.60 (−574.06, −79.15) for tofogliflozin and −185.72 (−217.29, −154.14) for pioglitazone. The results of the pairwise comparison are indicated by the MDs and 95% CI (Figure 12B). According to the results of SUCRA, tofogliflozin may be the most effective intervention to reduce the K-18 of patients, and the results of the SUCRA probability ranking are shown in Figure 12C.

### Invasive Tests

#### Fibrosis Score

With regard to a decrease in the fibrosis score, our NMA included six RCTs, reporting the administration of vitamin E, pioglitazone, and one SGLT2 inhibitor among 407 patients. There was no statistically significant association between the three drugs and a placebo, i.e., for pioglitazone −0.91 (95% CI: −1.82, 0.01), vitamin E −0.28 (−2.07, 1.51), and empagliflozin 0.02 (−1.76, 1.79). The results of the pairwise comparison are indicated by the SMDs and 95% CI (Figure 13B). According to the SUCRA results, pioglitazone may be the most effective intervention to reduce the fibrosis score of patients, and the results of the SUCRA probability ranking are shown in Figure 13C.

#### Resolution of NASH

With regard to an increase in the resolution of NASH, our NMA included seven RCTs, reporting the administration of vitamin E, pioglitazone, and one GLP-1 receptor agonist among 891 patients. Compared with a placebo, three included agents significantly increased the resolution, with OR of 6.43 (95% CI: 1.20, 34.41) for liraglutide, 3.55 (2.40, 5.28) for pioglitazone, and 2.27 (1.54, 3.35) for vitamin E. In addition, we found that pioglitazone increased the resolution compared with vitamin E, with an OR of 1.56 (1.03, 2.37). The results of the pairwise comparison are indicated by the OR and 95% CI (Figure 14B). According to the SUCRA results, liraglutide may be the most effective intervention to increase the NASH resolution of patients, and the results of the SUCRA probability ranking are shown in Figure 14C.

### Heterogeneity and Inconsistency Assessment

We tested the inconsistency of all comparisons. The results are presented in Supplementary Table 2. A *p* > 0.05 indicated no inconsistency among the direct and indirect comparisons, whereas a *p* < 0.05 indicated high inconsistency among the direct and indirect comparisons. The results showed that the direct and indirect inconsistencies of the included drugs were not high, except for one group in the analysis of ALT level (placebo vs. empagliflozin).

## Discussion

This study is a network meta-analysis designed to specifically evaluate the efficacy of vitamin E, pioglitazone, GLP-1 receptor agonists, and SGLT2 inhibitors for treating patients with NAFLD. The direct and indirect comparison results showed the evidence from recent RCTs. First, a placebo was used as a reference. δ-Tocotrienol was superior to placebo in decreasing the GGT level. Semaglutide, ipragliflozin, and pioglitazone induced a significantly higher decrease in the ALT level than a placebo. Semaglutide, pioglitazone, and dapagliflozin were superior to placebo in decreasing the AST level. Tofogliflozin and pioglitazone induced a significantly higher decrease in the K-18 level than a placebo. Liraglutide was superior to placebo in decreasing the CAP. Liraglutide, pioglitazone, and vitamin E induced a significantly higher increase in resolution of NASH compared with a placebo. As for pairwise comparisons, semaglutide and pioglitazone were superior to liraglutide in decreasing the ALT level. Semaglutide induced a significantly higher decrease in the ALT level than dulaglutide. Semaglutide was superior to empagliflozin, liraglutide, dulaglutide, and tofogliflozin in decreasing the AST level. Pioglitazone induced a significantly higher decrease in GGT level than ipragliflozin. δ-Tocotrienol was superior to liraglutide in decreasing the GGT level. Tofogliflozin and pioglitazone induced a significantly higher decrease in the K-18 level than dulaglutide. Pioglitazone was obviously superior to vitamin E in increasing the resolution of NASH. Furthermore, liraglutide treatment had the highest SUCRA ranking in decreasing the CAP and ELF score, and in increasing the resolution of NASH. Pioglitazone treatment had the highest SUCRA ranking in decreasing the LFC and fibrosis score. Tofogliflozin treatment had the highest SUCRA ranking in decreasing the K-18. Dapagliflozin treatment had the highest SUCRA ranking in decreasing the GGT level. Semaglutide treatment had the highest SUCRA ranking in decreasing the levels of ALT and AST. These findings indicate that more studies should be carried out to explore the potential of the above drugs in treating patients with NAFLD. Liraglutide, dulaglutide, and tofogliflozin were ranked last in the SUCRA ranking in reducing GGT, ALT, and AST, respectively. Additional large, high-quality studies are needed to confirm the efficacy of these drugs.

Serum biomarkers (such as ALT, AST, GGT) are the most common non-invasive tests for evaluating liver diseases and are usually used as clinical indicators of liver cell injury. However, they are often used as auxiliary indicators of the improvement of NASH, owing to their lack of specificity and sensitivity (45). According to mouse models of NASH, GLP-1 analogs reduces liver enzymes and oxidative stress, and also improve liver histology (46). We found that semaglutide (a GLP-1 receptor agonist) is superior to other drugs (including placebo) in reducing the ALT and AST levels. The same was reported by Cusi (47). K-18, a biomarker of hepatocyte apoptosis, was also analyzed in this NMA. The results showed that tofogliflozin and pioglitazone could obviously reduce the level of K-18.

In view of the fact that liver biopsy is related to patients' severe advents, some non-invasive alternative indicators have been suggested in different countries and regions, such as tests used to quantify the degree of liver steatosis (e.g., MRI–PDFF, L/S ratio, and CAP score) and tests used to judge the severity of liver fibrosis (e.g., FIB-4 index, Pro-3, LSM, APRI, and ELF score) (48). This NMA reported on CAP, ELF score, and LFC. CAP evaluates steatosis based on shear-wave propagation, and its performance has been supported by several biopsy-controlled clinical studies (27, 32, 43). Liver fat measured by MRI–PDFF or H-MRS quantitatively explores LFC through free radiation mode (31, 34, 40). The ELF score has mostly been determined in cohort studies with a high prevalence of advanced fibrosis and has been tested in various cross-sectional studies and clinical trials (11). The results of this NMA showed that the liraglutide treatment had the highest ranking in decreasing CAP and ELF scores, while the pioglitazone treatment had the highest ranking in decreasing the LFC and fibrosis score. Previous studies have reported that GLP-1 analogs can act directly on human hepatocytes *in vitro*, reducing steatosis by decreasing *de novo* lipogenesis and increasing fatty acid oxidation (49–51). Pioglitazone can improve insulin sensitivity and transfer fat accumulation from central to subcutaneous regions (37). These results may explain the mechanism of clinical efficacy of the above drugs. FIB-4 index, Pro-3, L/S ratio, APRI, and LSM were not analyzed in this study because large, high-quality studies are still needed.

The gold standard method for diagnosing NAFLD is liver biopsy, which reveals the main histopathological features of NAFLD, such as steatosis, hepatocellular ballooning, lobular inflammation, and fibrosis (45). These findings can be used to score histology and judge the activity of NAFLD. Although there are some objective problems, such as difficult puncture techniques and errors in sample acquisition, liver biopsy is still the primary method of diagnosis, as there are no accurate non-invasive tests. Due to the limitations of the included literature, this NMA only analyzed the fibrosis score and the resolution of histological features that met the diagnostic criteria of NASH. According to the results of SUCRA, we found that liraglutide could be the most effective intervention to achieve resolution of NASH, followed by pioglitazone and vitamin E. Vitamin E is thought to improve NASH by reducing oxidative stress of hepatocytes and then reduce liver injury and inflammation, while pioglitazone can improve insulin sensitivity (52–54). Previous studies have found that liraglutide can improve body weight and blood glucose control, which may have a beneficial effect on the risk of cardiovascular disease and premature death in patients with NASH in the future. However, evidence for directly improving the hepatic histology of NASH remains to be explored (42).

There are some other off-label drugs used in the treatment of NAFLD worldwide, such as omega-3 fatty acids, metformin, and statins. However, these drugs have shown no discernible histological benefit on NASH yet (11). Furthermore, obeticholic acid, a potent farnesoid × receptor agonist, has shown a side effect (pruritus) that has reduced the possibility of its conditional approval (11). In addition, silybin or its complexes might play a potential role in the treatment of NAFLD patients. A preliminary observational study in 2006 (55) showed that treatment with the silybin–vitamin E–phospholipid complex for 6 months was able to improve the ultrasound appearance of the “bright” liver, the levels of liver enzymes, homeostatic model assessment for insulin resistance (HOMA-IR), and serum indices of hepatic fibrosis; these preliminary results were then also confirmed in a phase III study on 180 patients (55). In other words, silybin and its complexes are areas worthy of further study in the future. Our study did not consider some current ideas based on specific genetic background (including polymorphisms in key genes in the pathogenesis of NAFLD) that could potentially negatively influence or decrease responses to certain therapeutic regimens (including silybin-based regimens) (56), so these ideas still need to be explored.

Our study has several limitations. First, there was a small number of open-label studies we could include (17, 26, 30, 31). Second, the basic characteristics of some studies mentioned that several patients had temporarily used other drugs (e.g., to avoid serious endocrine imbalance). Third, the drugs mentioned in this review were in phase II or III clinical trials at that time, so the accuracy of the results of indirect comparison sounded bad with lacking direct comparison. Therefore, our results need to be verified in multicenter and large-scale studies; especially those with long-term and complete follow-up.

## Conclusion

The network meta-analysis provided evidence for the efficacy of vitamin E, pioglitazone, SGLT2 inhibitors, and GLP-1 receptor agonists in NAFLD patients. In order to find the best guide-level drugs, it is necessary to include more RCT experiments with these off-label drugs, so that patients and clinicians can make optimal decisions together.

## Data Availability Statement

The original contributions presented in the study are included in the article/Supplementary Material, further inquiries can be directed to the corresponding author/s.

## Author Contributions

QL, RW, and YC: research idea, study design, and data analyses/interpretation. YC and QZ: data acquisition and statistical analyses. QL, RW, YC, and QZ: supervision or mentorship. All author contributed important intellectual content during manuscript drafting or revision and accepts accountability for the overall work by ensuring that questions on the accuracy or integrity of any portion of the work are appropriately investigated and resolved.

## Funding

This study was supported by the National Major Scientific and Technological Special Project for ‘‘Significant New Drugs Development'' (grant no. 2017ZX09304019), National Natural Science Foundation of China (no. 81774278).

## Conflict of Interest

The authors declare that the research was conducted in the absence of any commercial or financial relationships that could be construed as a potential conflict of interest.

## Publisher's Note

All claims expressed in this article are solely those of the authors and do not necessarily represent those of their affiliated organizations, or those of the publisher, the editors and the reviewers. Any product that may be evaluated in this article, or claim that may be made by its manufacturer, is not guaranteed or endorsed by the publisher.

## Abbreviations

NAFLD: non-alcoholic fatty liver disease
CAP: controlled attenuation parameter
LFC: liver fat content
ELF: enhanced liver fibrosis
K-18: keratin-18 (K-18) M30 fragment
ALT: alanine aminotransferase
AST: aspartate aminotransferase
FDA: Food and Drug Administration
EMA: European Medicines Agency
GGT: gamma-glutamyl transferase
GLP-1: glucagon-like peptide-1
HCC: hepatocellular carcinoma
HOMA-IR: homeostatic model assessment for insulin resistance
PPARγ: peroxisome proliferator-activated receptor γ
SGLT2: sodium-glucose cotransporter-2
RCT: Randomized controlled trial
NICE: the United Kingdom's National Institute for Health and Clinical Excellence
NASH: non-alcoholic steatohepatitis.
