# Supplementary material for: Efficacy of Off-Label Therapy for Non-alcoholic Fatty Liver Disease in Improving Non-invasive and Invasive Biomarkers: A Systematic Review and Network Meta-Analysis of Randomized Controlled Trials
Source: Front Med (Lausanne). 2022 Feb 25;9:793203. doi: 10.3389/fmed.2022.793203 (PMC8914474; doi:10.3389/fmed.2022.793203)
Supplement: Supplementary file 1 [file Table_1.docx]

| **Table 1. Characteristics of the included studies in the meta-analysis** | | | | | | |  |
| --- | --- | --- | --- | --- | --- | --- | --- |
| **Study** | **Inclusion Criteria** | **Sample Size(T/no-T)** | **No. of**  **patient**  **(% of male)** | **Age, (yr)**  **mean ± SD** | **Treatment**  **Duration** | **Intervention** | **Outcome Index** |
| ^7^J. H. Hoofnagle 2013 | patients with non-alcoholic steatohepatitis | 71/68 | I：41%  C：44% | I：46±12  C：45±12 | 96 weeks | I：vitamin E,800IU/day,orally  C：Placebo | Resolution of NASH |
| ^14^Golnaz Ekhlasi 2017 | NAFLD patients | 15/15 | I：80%  C：80% | I：25-64  C：25-64 | 8 weeks | I：Alphatocopherol,400IU/day,orally  C：Placebo | AST |
| ^15^Amalia Gastaldelli 2021 | steatohepatitis in patients  with NASH | 17/18 |  | I：51 ± 2  C：51±3 | 6 months | I：Pioglitazone,45mg/day orally  C：Placebo | liver fat content,  AST,Fibrosis score |
| ^21^Jee Fu 2021 | nonalcoholic steatohepatitis patients | 43/47 | I：62.8%  C：83.0% | I：43.9±13.7  C：43.8±11.9 | 24 weeks | I：Pioglitazone,30mg/day orally  C：Placebo | liver fat content,Resolution of NASH,ALT,AST,  Fibrosis score |
| ^22^Muhammad Amjad Pervez 2020 | patients with nonalcoholic fatty liver disease | 35/36 | I：54.3%  C：41.7% | I:44.94± 8.50  C：43.91± 8.40 | 24 weeks | I：δ-tocotrienol,300mg tid orally  C：Placebo | K-18,ALT,AST,GGT |
| ^23^GURUPRASAD 2008 | Nondiabetic Subjects With Nonalcoholic Steatohepatitis | 37/37 | I：70%  C：51% | I：52 (28–71)  C：55 (27–73) | 12 months | I：Pioglitazone,30mg/day orally  C：Placebo | ALT,GGT |
| ^24^Louise Vedtofte 2020 | Women with Nonalcoholic Fatty Liver Disease and Prior Gestational Diabetes Mellitus | 37/45 |  | I: 38.8(34.3;40.7)  C：38.3(35.5;41.2) | 12 months | I：Liraglutide,1.8mg/day,by subcutaneous injection  C：Placebo | CAP,ALT,AST,GGT |
| ^25^Kyu Yong Cho 2020 | participants with NAFLD | 27/26 | I：55.6%  C：50.0% | I：63.5 ± 7.1  C：63.4 ±10.2 | 24 weeks | I：Dapagliflozin  C：Pioglitazone | ALT,AST,GGT |
| ^12^Renata Belfort 2006 | patients with impaired glucose tolerance or type 2 diabetes  and NASH | 26/21 | I：53%  C：33% | I：51±7  C：51±10 | 6 months | I：Pioglitazone 45mg/day orally  C：Placebo | liver fat content,ALT,AST,Fibrosis score |
| ^26^Arun J2010 | adults with nonalcoholic steatohepatitis and without diabetes | 80(P)/84(V)/83(placebo) | I(P)：26%  I(V):26%  C：30% | I(P)：47.0±12.6  I(V):46.6±12.1  C：45.4±11.2 | 96 weeks | I(P)：Pioglitazone 30mg/day orally  I(V): vitamin E 800IU/day orally  C： Placebo | Resolution of NASH |
| ^27^P.N. Newsome 2021 | patients with biopsy  confirmed NASH and liver fibrosis of stage F1, F2, or F3. | 80(0.1mg)/78(0.2mg)/82(0.4mg)/80(placebo) | I(0.1mg)：36%  I(0.2mg):33%  I(0.4mg):42%  C：45% | I(0.1mg)：55.2±10.9  I(0.2mg):58.1±9.9  I(0.4mg):54.3±10.2  C：52.4±10.8 | 72 weeks | I(0.1mg)：Semaglutide 0.1mg once daily,by subcutaneous injection  I(0.2mg): Semaglutide 0.2mg once daily，by subcutaneous injection  I(0.4mg): Semaglutide 0.4mg once daily，by subcutaneous injection  C：Placebo | ALT,AST |
| ^20^Samer Gawrieh 2021 | adults with biopsy-proven NASH without cirrhosis or diabetes | 78(P)/83(V)/82(placebo) | I(P)：41%  I(V):39%  C：43% | I(P)：47.3 (12.6)  I(V):46.8 (12.1)  C：45.5 (11.2) | 16/48/96 weeks | I(P)： Pioglitazone 30mg/day orally  I(V): vitamin E 800IU/day  C： Placebo | ELF |
| ^28^Jan W. Eriksson 2018 | non-alcoholic fatty liver disease in people with type 2 diabetes | 21/21 | I： 68%  C： 80% | I： 65.0 (6.5)  C：65.6 (6.1) | 12 weeks | I： Dapagliflozin 10 mg/day  C：Placebo | liver fat content |
| ^29^Haleh 2021 | Patients with Non-Alcoholic Fatty Liver Disease and Type 2 Diabetes | 35(E)/34(P)/37(placebo) | I(E)：42.9%  I(P): 50.0%  C： 37.8% | I(E)：50.5 ± 8.4  I(P): 52.5 ± 7.9  C： 51.8 ± 7.8 | 24 weeks | I(E)： Empagliflozin 10mg/day orally  I(P): Pioglitazone 30mg/day orally  C： Placebo | CAP,ALT,AST, |
| ^30^Daisuke Ito 2017 | Nonalcoholic Fatty Liver Disease  in Patients With Type 2 Diabetes | 32/34 | I： 44%  C： 53% | I：57.3 ± 12.1  C：59.1 ± 9.8 | 24 weeks | I：Ipragliflflozin 50mg/day orally  C：Pioglitazone 15–30 mg/day orally | ALT,AST,GGT |
| ^13^Fernando Bril 2019 | Nonalcoholic Steatohepatitis in Patients With Type 2 Diabetes | 36/32 | I： 91%  C： 93% | I：60 ± 9  C：57 ± 11 | 18 months | I：Vitamin E 400 IU b.i.d  C：Placebo | Resolution of NASH,Fibrosis score |
| ^31^Sabine Kahl 2019 | Type 2 Diabetes wiht abnormal ALT,AST(exclusion criteria included Liver disease other than NAFLD) | 42/42 | I： 69%  C： 69% | I：62.7 ± 7.0  C：61.5 ± 10.0 | 24 weeks | I： Empagliflozin 25 mg/day orally  C： Placebo | liver fat content |
| ^32^Mark L 2020 | Nonalcoholic Steatohepatitis in Patients With Type 2 Diabetes | 54/51 | I: 44%  C：57% | I:58.7 (7.8)  C：56.6 (8.9) | 26w | I:Dulaglutide 1.5 mg/week,by subcutaneously injected  C： Placebo | K-18,ALT,AST |
| ^33^Tomoe 2020 | nonalcoholic fatty liver disease in cases with type 2 diabetes | 32/33 | I：46.9%  C：45.5% | I：58.7±1.6  C：59.0±1.9 | 28 weeks | I： Dapagliflozin 5 mg/day orally  C： Pioglitazone 7.5-15mg/day | Resolution of NASH |
| ^34^Mark D 2021 | Individuals with biopsy-confirmed NASH | 72(V)/62(P)/67(placebo) | I(V)：37.5%  I(P): 40.3%  C： 44.8% | I(V)：47.4 (12.2)  I(P):47.6 (12.7)  C：47.0 (11.5) | 96 weeks | I(V)：vitamin-E 800 IU/day  I(P): Pioglitazone 30mg/day  C： Placebo | I(V)：47.4 (12.2)  I(P):47.6 (12.7)  C：47.0 (11.5) |
| ^35^Kenneth Cusi 2016 | Nonalcoholic  Steatohepatitis and Prediabetes or Type 2 Diabetes Mellitus | 50/51 | I： 72%  C： 69% | I：52 (10)  C：49 (11) | 18 months | I：Pioglitazone 45mg/day orally  C：Placebo | Resolution of NASH,K-18,ALT,AST,Fibrosis score |
| ^36^Susrichit 2021 | type 2 diabetes patients with non-alcoholic fatty liver disease | 18/20 | I： 28%  C： 35% | I： 57.0 ± 6.9  C： 61.2 ± 7.2 | 12 weeks | I： Dapagliflozin 10mg/day orally  C： Placebo | ALT,AST |
| ^37^Mark M 2016 | patients with type 2 diabetes  With abnormal liver function and Hepatic histologic | 17/17 | I： 70.6%  C： 76.5% | I：60.8 ± 1.8  C： 65.8 ± 1.4 | 12 weeks | I：Liraglutide 1.8mg/day  C：Placebo | liver fat content,ALT,AST,GGT |
| ^38^Maurice B 2019 | individuals with type 2 diabetes With abnormal liver function | 23/26 | I： 61%  C： 58% | I：60 ± 6  C： 59 ± 7 | 26 weeks | I：Liraglutide 1.8mg/day,  C：Placebo | ALT,AST,GGT |
| ^39^Matthew J 2015 | Patients with non-alcoholic  steatohepatitis | 26/26 | I： 69%  C： 50% | I：50 (11)  C： 52 (12) | 48 weeks | I：Liraglutide 1.8mg/day,by subcutaneous injections  C： Placebo | ELF,Resolution of NASH,K-18,ALT,AST,GGT |
| ^40^Hoda Taheri 2020 | Patients With Non-Alcoholic Fatty Liver Disease Without Diabetes | 43/47 | I： 46.8%  C： 12.2% | I： 43.8 (9.7)  C： 44.1 (9.3) | 24 weeks | I：Empagliflozin 10mg/day orally  C：Placebo | CAP,ALT,AST,Fibrosis score |
| ^41^Masato 2021 | non-alcoholic fatty liver disease patients with type 2  diabetes mellitus | 21/19 | I： 61.9%  C： 42.1% | I：58.4±12.2  C：58.8±8.1 | 24 weeks | I：Tofogliflozin 20mg/day orally  C：Pioglitazone 15–30mg/day orally | liver fat content,K-18,ALT,AST,GGT |

| **Table 2. The Inconsistency** | | | | | | | | |
| --- | --- | --- | --- | --- | --- | --- | --- | --- |
| Nodes | Direct effect | | Indirect effect | | Difference | | | tau |
|  | Coef. | Std.Err. | Coef. | Std.Err. | Coef. | Std.Err. | P>ΙzΙ |  |
| Liver Fat Content(LFC) | | | | | | | | |
| Placebo,Pioglitazone | -21.38515 | 15.28939 | -1.890001 | 200.021 | -19.49515 | 200.6045 | 0.923 | 24.98394 |
| Pioglitazone,Tofogliflozin | 3.420002 | 25.0688 | 42.4103 | 400.4264 | -38.9903 | 401.2121 | 0.923 | 24.98394 |
| Resolution of NASH | | | | | | | | |
| Placebo,Pioglitazone | 1.259657 | 0.2108797 | 1.375455 | 0.7978914 | -0.1157973 | 0.8343372 | 0.890 | 6.72e-13 |
| Placebo,vitaminE | 0.8089294 | 0.2059187 | 0.992303 | 0.832807 | -0.1833736 | 0.8668491 | 0.832 | 1.59e-12 |
| Pioglitazone,vitaminE | -0.4428122 | 0.2360951 | -0.4660141 | 0.4929171 | 0.023202 | 0.5464998 | 0.966 | 4.95e-13 |
| ALT | | | | | | | | |
| Placebo,Pioglitazone | -17.48675 | 4.67908 | -5.135314 | 8.939829 | -12.35144 | 10.0897 | 0.221 | 7.147843 |
| Placebo,Dapagliflozin | -3.5 | 7.778103 | -16.39764 | 7.575229 | 12.89764 | 10.85739 | 0.235 | 7.166311 |
| Placebo, Empagliflozin | -2.773606 | 5.346377 | -37.20818 | 15.63891 | 34.43458 | 16.6013 | 0.038 | 6.292798 |
| Pioglitazone,Dapagliflozin | 0.7311669 | 6.050003 | 13.63592 | 9.028617 | -12.90475 | 10.85841 | 0.235 | 7.166551 |
| Pioglitazone,Empagliflozin | 0.5839762 | 7.812168 | 18.37827 | 9.161702 | -17.79429 | 12.03692 | 0.139 | 6.924722 |
| Pioglitazone,Tofogliflozin | 10.5 | 11.51941 | 29.35035 | 1783.746 | -18.85035 | 1783.76 | 0.992 | 7.134368 |
| Pioglitazone,Ipragliflflozin | -2.5 | 7.198366 | 29.84408 | 235.0664 | -32.34408 | 235.1771 | 0.891 | 7.140353 |
| AST | | | | | | | | |
| Placebo,Pioglitazone | -9.203623 | 2.670488 | -1.215403 | 6.149129 | -7.988221 | 6.706721 | 0.234 | 4.433147 |
| Placebo,Dapagliflozin | -3.5 | 5.837402 | -10.16725 | 4.778759 | 6.667247 | 7.543991 | 0.377 | 4.555697 |
| Placebo,Empagliflozin | -3.139815 | 3.394077 | -20.33315 | 9.936452 | 17.19333 | 10.36892 | 0.097 | 4.133008 |
| Pioglitazone,Dapagliflozin | -1.369137 | 3.967685 | 5.301657 | 6.415876 | -6.670795 | 7.544122 | 0.377 | 4.555748 |
| Pioglitazone,Empagliflozin | -2.318558 | 5.041876 | 8.900207 | 5.215817 | -11.21876 | 7.182433 | 0.118 | 4.188361 |
| Pioglitazone,Tofogliflozin | 12.1 | 6.891446 | 15.30748 | 1043.731 | -3.207478 | 1043.742 | 0.998 | 4.457997 |
| Pioglitazone,Ipragliflflozin | -1 | 4.508434 | 15.89702 | 168.9043 | -16.89702 | 168.9654 | 0.920 | 4.459483 |
| Gamma glutamyl transferase(GGT) | | | | | | | | |
| Placebo,Pioglitazone | -115.7 | 82.05801 | -2.28139 | 158.4347 | -113.4186 | 178.4239 | 0.525 | 2.81e-09 |
| Pioglitazone,Dapagliflozin | -0.8797524 | 3.240056 | 190.6035 | 769.262 | -191.4833 | 769.2672 | 0.803 | 1.26e-11 |
| Pioglitazone,Tofogliflozin | 29.4 | 10.05467 | 182.7638 | 2002.736 | -153.3638 | 2002.768 | 0.939 | 1.96e-06 |
| Pioglitazone,Ipragliflflozin | 5.1 | 1.378645 | 219.8488 | 389.0556 | -214.7488 | 389.0583 | 0.581 | 4.81e-07 |

**Search strategies**

**Pubmed**

((((((((((((Non-alcoholic Fatty Liver Disease[Title/Abstract]) OR (Non alcoholic Fatty Liver Disease[Title/Abstract])) OR (NAFLD[Title/Abstract])) OR (Nonalcoholic Fatty Liver Disease[Title/Abstract])) OR (Nonalcoholic Fatty Liver[Title/Abstract])) OR (Nonalcoholic Fatty Livers[Title/Abstract])) OR (Nonalcoholic Steatohepatitis[Title/Abstract])) OR (Nonalcoholic Steatohepatitides[Title/Abstract]))) OR ("Non-alcoholic Fatty Liver Disease"[Mesh])) AND ((((((((compare[title/abstract] OR compared [title/abstract]) OR comparison[title/abstract]) OR comparative[title/abstract]) OR comparing[title/abstract]) OR versus[title/abstract]) OR vs[title/abstract]))))) AND ((((((((Randomized Controlled Trial[ptyp] OR controlled clinical trial[ptyp]) OR randomized[title/abstract]) OR randomised[title/abstract]) OR randomly[title/abstract]) OR trial[title/abstract]) OR phase[title/abstract])))) AND ((((treatment[Title/Abstract]) OR (therapy[Title/Abstract]))) OR ((((((((((((((((((((((((((((((((((Sodium-Glucose Transporter 2 Inhibitors[Title/Abstract]) OR (Sodium Glucose Transporter 2 Inhibitors[Title/Abstract])) OR (Sodium-Glucose Transporter 2 Inhibitor[Title/Abstract])) OR (Sodium Glucose Transporter 2 Inhibitor[Title/Abstract])) OR (SGLT-2 Inhibitors[Title/Abstract])) OR (SGLT 2 Inhibitors[Title/Abstract])) OR (Gliflozins[Title/Abstract])) OR (SGLT2 Inhibitors[Title/Abstract])) OR (Gliflozin[Title/Abstract])) OR (SGLT-2 Inhibitor[Title/Abstract])) OR (SGLT 2 Inhibitor[Title/Abstract])) OR (SGLT2 Inhibitor[Title/Abstract])) OR ("Sodium-Glucose Transporter 2 Inhibitors"[Mesh])) OR ((((((Sodium-Glucose Transporter 2 Inhibitors[Title/Abstract]) OR (Invokana[Title/Abstract])) OR (Canagliflozin Hemihydrate[Title/Abstract])) OR (Canagliflozin, Anhydrous[Title/Abstract])) OR (1-(Glucopyranosyl)-4-methyl-3-(5-(4-fluorophenyl)-2-thienylmethyl)benzene - T777973[Title/Abstract])) OR ("Sodium-Glucose Transporter 2 Inhibitors"[Mesh]))) OR (((((((((dapagliflozin[Title/Abstract]) OR ((2S,3R,4R,5S,6R)-2-(4-chloro-3-(4-ethoxybenzyl)phenyl)-6- (hydroxymethyl)tetrahydro-2H-pyran-3,4,5-triol[Title/Abstract])) OR (Farxiga[Title/Abstract])) OR (Forxiga[Title/Abstract])) OR (2-(3-(4-ethoxybenzyl)-4-chlorophenyl)-6-hydroxymethyltetrahydro-2H-pyran-3,4,5-triol[Title/Abstract])) OR (BMS 512148[Title/Abstract])) OR (BMS512148[Title/Abstract])) OR (BMS-512148[Title/Abstract])) OR ("dapagliflozin" [Supplementary Concept]))) OR (((((((5-(4-chloro-3-(4-ethoxybenzyl)phenyl)-1-hydroxymethyl-6,8-dioxabicyclo(3.2.1)octane-2,3,4-triol[Title/Abstract]) OR (Steglatro[Title/Abstract])) OR (PF 04971729[Title/Abstract])) OR (PF04971729[Title/Abstract])) OR (PF-04971729[Title/Abstract])) OR (ertugliflozin[Title/Abstract])) OR ("ertugliflozin" [Supplementary Concept]))) OR (((((((1S)-1,5-anhydro-1-(3-(1-benzothiophen-2-ylmethyl)-4-fluorophenyl)-D-glucitol[Title/Abstract]) OR (Suglat[Title/Abstract])) OR (ASP1941[Title/Abstract])) OR (ASP-1941[Title/Abstract])) OR (ipragliflozin[Title/Abstract])) OR ("ipragliflozin" [Supplementary Concept]))) OR (((((((luseogliflozin[Title/Abstract]) OR (Lusefi[Title/Abstract])) OR (TS 071[Title/Abstract])) OR (TS071 cpd[Title/Abstract])) OR (TS-071[Title/Abstract])) OR (1,5-anhydro-1-(5-(4-ethoxybenzyl)-2-methoxy-4-methylphenyl)-1-thioglucitol[Title/Abstract])) OR ("1,5-anhydro-1-(5-(4-ethoxybenzyl)-2-methoxy-4-methylphenyl)-1-thioglucitol" [Supplementary Concept]))) OR (((4-(4-isopropoxybenzyl)-1-isopropyl-5-methyl-1H-pyrazol-3-yl 6-O-(ethoxycarbonyl)-beta-D-glucopyranoside[Title/Abstract]) OR (remogliflozin etabonate[Title/Abstract])) OR ("remogliflozin etabonate" [Supplementary Concept]))) OR (((((sotagliflozin[Title/Abstract]) OR (LX4211[Title/Abstract])) OR (LX-4211[Title/Abstract])) OR ((2S,3R,4R,5S,6R)-2-(4-chloro-3-(4-ethoxybenzyl)phenyl)-6-(methylthio)tetrahydro-2H-pyran-3,4,5-triol[Title/Abstract])) OR ("(2S,3R,4R,5S,6R)-2-(4-chloro-3-(4-ethoxybenzyl)phenyl)-6-(methylthio)tetrahydro-2H-pyran-3,4,5-triol" [Supplementary Concept]))) OR ((sergliflozin[Title/Abstract]) OR ("sergliflozin" [Supplementary Concept]))) OR ((((((((tofogliflozin hydrate[Title/Abstract]) OR (CSG452[Title/Abstract])) OR (tofogliflozin anhydrous[Title/Abstract])) OR (Apleway[Title/Abstract])) OR (Deberza[Title/Abstract])) OR (tofogliflozin[Title/Abstract])) OR (6-((4-ethylphenyl)methyl)-3',4',5',6'-tetrahydro-6'-(hydroxymethyl)spiro(isobenzofuran-1(3H),2'-(2H)pyran)-3',4',5'-triol[Title/Abstract])) OR ("6-((4-ethylphenyl)methyl)-3',4',5',6'-tetrahydro-6'-(hydroxymethyl)spiro(isobenzofuran-1(3H),2'-(2H)pyran)-3',4',5'-triol" [Supplementary Concept]))) OR (((((((1-chloro-4-(glucopyranos-1-yl)-2-(4-(tetrahydrofuran-3-yloxy)benzyl)benzene[Title/Abstract]) OR (BI 10773[Title/Abstract])) OR (BI10773[Title/Abstract])) OR (BI-10773[Title/Abstract])) OR (Jardiance[Title/Abstract])) OR (empagliflozin[Title/Abstract])) OR ("empagliflozin" [Supplementary Concept]))) OR (((((((((Glucagon Like Peptide 1 Receptor[Title/Abstract]) OR (GLP-1R Receptor[Title/Abstract])) OR (GLP 1R Receptor[Title/Abstract])) OR (GLP1R Protein[Title/Abstract])) OR (GLP-1 Receptor[Title/Abstract])) OR (GLP 1 Receptor[Title/Abstract])) OR (GLP1R Receptor[Title/Abstract])) OR (Glucagon-Like Peptide-1 Receptor[Title/Abstract])) OR ("Glucagon-Like Peptide-1 Receptor"[Mesh]))) OR ((((((((((Bydureon[Title/Abstract]) OR (ITCA 650[Title/Abstract])) OR (AC 2993 LAR[Title/Abstract])) OR (Exendin-4[Title/Abstract])) OR (Ex4 Peptide[Title/Abstract])) OR (Exendin 4[Title/Abstract])) OR (Byetta[Title/Abstract])) OR (AC 2993[Title/Abstract])) OR (Exenatide[Title/Abstract])) OR ("Exenatide"[Mesh]))) OR ((((((((Victoza[Title/Abstract]) OR (Saxenda[Title/Abstract])) OR (NN 2211[Title/Abstract])) OR (2211, NN[Title/Abstract])) OR (NN2211[Title/Abstract])) OR (NN-2211[Title/Abstract])) OR (Liraglutide[Title/Abstract])) OR ("Liraglutide"[Mesh]))) OR ((((((((((((((DES-38-proline-exendine-4 (Heloderma suspectum)-(1-39)-peptidylpenta-l-lysyl-l-lysinamide[Title/Abstract]) OR (Adlyxin[Title/Abstract])) OR (AQVE-10010[Title/Abstract])) OR (ZP10A peptide[Title/Abstract])) OR (ZP 10[Title/Abstract])) OR (ZP-10[Title/Abstract])) OR (Lyxumia[Title/Abstract])) OR (AVE 010[Title/Abstract])) OR (AVE-010[Title/Abstract])) OR (AVE 0010[Title/Abstract])) OR (AVE0010[Title/Abstract])) OR (AVE-0010[Title/Abstract])) OR (lixisenatide[Title/Abstract])) OR ("lixisenatide" [Supplementary Concept]))) OR (((((Eperzan[Title/Abstract]) OR (Tanzeum[Title/Abstract])) OR (albiglutide[Title/Abstract])) OR (rGLP-1 protein[Title/Abstract])) OR ("rGLP-1 protein" [Supplementary Concept]))) OR ((((((LY 2189265[Title/Abstract]) OR (LY-2189265[Title/Abstract])) OR (LY2189265[Title/Abstract])) OR (Trulicity[Title/Abstract])) OR (dulaglutide[Title/Abstract])) OR ("dulaglutide" [Supplementary Concept]))) OR ((((rybelsus[Title/Abstract]) OR (Ozempic[Title/Abstract])) OR (semaglutide[Title/Abstract])) OR ("semaglutide" [Supplementary Concept]))) OR ((((((((((((5-(4-(2-(5-Ethyl-2-pyridyl)ethoxy)benzyl)-2,4-thiazolidinedione[Title/Abstract]) OR (U 72107A[Title/Abstract])) OR (U72,107A[Title/Abstract])) OR (U-72107A[Title/Abstract])) OR (U72107A[Title/Abstract])) OR (AD 4833[Title/Abstract])) OR (AD-4833[Title/Abstract])) OR (AD4833[Title/Abstract])) OR (Pioglitazone Hydrochloride[Title/Abstract])) OR (Actos[Title/Abstract])) OR (Pioglitazone[Title/Abstract])) OR ("Pioglitazone"[Mesh]))) OR (((((((((((((((vitamin E[MeSH Terms]) OR (alpha tocopherol[Title/Abstract])) OR (vitamin E[Title/Abstract])) OR (vitamin*[Title/Abstract])) OR (Tocopherols[MeSH Terms])) OR (Tocopherols[Title/Abstract])) OR (Tocopherol[Title/Abstract])) OR (Tocovital[Title/Abstract])) OR (tocotrienol[Title/Abstract])) OR (Vitazell[Title/Abstract])) OR (Detulin[Title/Abstract])) OR (Embial[Title/Abstract])) OR (Evion[Title/Abstract])) OR (Ephynal[Title/Abstract]))))) OR (((((((((((((((((((((((((((((((((((((((((((((((((((((((((((((((((((((((((((Tocopherol[Title/Abstract]) OR (Tocovital[Title/Abstract])) OR (Uno-Vit[Title/Abstract])) OR (Uno Vit[Title/Abstract])) OR (UnoVit[Title/Abstract])) OR (Vita-Plus E[Title/Abstract])) OR (Vita Plus E[Title/Abstract])) OR (VitaPlus E[Title/Abstract])) OR (Vitagutt Vitamin E[Title/Abstract])) OR (Vitamin E, Vitagutt[Title/Abstract])) OR (Vitamin E AL[Title/Abstract])) OR (Vitamin E Natur[Title/Abstract])) OR (Vitamin E Sanum[Title/Abstract])) OR (Vitamin E Suspension[Title/Abstract])) OR (Vitamin-E Dragees[Title/Abstract])) OR (Vitamin E Dragees[Title/Abstract])) OR (Vitamin-E EVI-MIRALE[Title/Abstract])) OR (Vitamin E EVI MIRALE[Title/Abstract])) OR (VitaminE EVIMIRALE[Title/Abstract])) OR (Vitamine E GNR[Title/Abstract])) OR (Vitazell[Title/Abstract])) OR (Detulin[Title/Abstract])) OR (E Vitamin E[Title/Abstract])) OR (Vitamin E-mp[Title/Abstract])) OR (Vitamin E mp[Title/Abstract])) OR (Vitamin Emp[Title/Abstract])) OR (E-Mulsin[Title/Abstract])) OR (E Mulsin[Title/Abstract])) OR (E-Vicotrat[Title/Abstract])) OR (E Vicotrat[Title/Abstract])) OR (E-Vitamin-Ratiopharm[Title/Abstract])) OR (Ecoro[Title/Abstract])) OR (Elex Verla[Title/Abstract])) OR (Embial[Title/Abstract])) OR (Evion[Title/Abstract])) OR (Ephynal[Title/Abstract])) OR (Eplonat[Title/Abstract])) OR (Equivit E[Title/Abstract])) OR (EUNOVA Vitamin E[Title/Abstract])) OR (Antioxidans E-Hevert[Title/Abstract])) OR (Eusovit[Title/Abstract])) OR (Hydrovit E[Title/Abstract])) OR (Micorvit E[Title/Abstract])) OR (Malton E[Title/Abstract])) OR (Mowivit Vitamin E[Title/Abstract])) OR (Puncto E[Title/Abstract])) OR (Richtavit E[Title/Abstract])) OR (Sanavitan S[Title/Abstract])) OR (Dal-E[Title/Abstract])) OR (Dal E[Title/Abstract])) OR (Abortosan[Title/Abstract])) OR (Aquasol E[Title/Abstract])) OR (Auxina E[Title/Abstract])) OR (Bio E[Title/Abstract])) OR (Biopto-E[Title/Abstract])) OR (Biosan[Title/Abstract])) OR (Unique EE-ferol[Title/Abstract])) OR (Togasan Vitamin E[Title/Abstract])) OR (Tocopherol Bayer[Title/Abstract])) OR (VitaE[Title/Abstract])) OR (Vita E[Title/Abstract])) OR (Vita-E[Title/Abstract])) OR (Vit. E Stada[Title/Abstract])) OR (Vit E hydrosol[Title/Abstract])) OR (Vibolex[Title/Abstract])) OR (Tocopharm[Title/Abstract])) OR (Tocopa[Title/Abstract])) OR (Tocolion[Title/Abstract])) OR (Spondyvit[Title/Abstract])) OR (Dermorelle[Title/Abstract])) OR (Davitamon[Title/Abstract])) OR (Bioweyxin[Title/Abstract])) OR (Lasar[Title/Abstract])) OR (Tocopherols[Title/Abstract])) OR ("Tocopherols"[Mesh]))) OR (((Tocotrienol[Title/Abstract]) OR (Tocotrienols[Title/Abstract])) OR ("Tocotrienols"[Mesh]))))

**Embase**

(('nonalcoholic fatty liver'/exp OR 'nonalcoholic fatty liver':ab,ti OR 'nafld (nonalcoholic fatty liver disease)':ab,ti OR 'non alcoholic fatty liver disease':ab,ti OR 'non alcoholic hepato-steatosis':ab,ti OR 'non alcoholic hepatosteatosis':ab,ti OR 'non alcoholic liver steatosis':ab,ti OR 'non alcoholic steatotic hepatopathy':ab,ti OR 'non-alcoholic fatty liver':ab,ti OR 'non-alcoholic fatty liver disease':ab,ti OR 'non-alcoholic fld':ab,ti OR 'non-alcoholic hepatic steatosis':ab,ti OR 'nonalcoholic fatty liver disease':ab,ti OR 'nonalcoholic fld':ab,ti OR 'nonalcoholic hepatic steatosis':ab,ti OR 'nonalcoholic hepatosteatosis':ab,ti OR 'nonalcoholic liver steatosis':ab,ti) AND ('compare':ab,ti OR'compared':ab,ti OR 'comparison':ab,ti OR 'comparative':ab,ti OR 'comparing':ab,ti OR 'versus':ab,ti OR 'vs':ab,ti) AND ('randomized controlled trial':it OR 'controlled clinical trial':it OR 'randomized controlled trial':ab,ti OR 'controlled clinical trial':ab,ti OR 'randomized':ab,ti OR 'randomised':ab,ti OR 'randomly':ab,ti OR 'trial':ab,ti OR 'phase':ab,ti)) AND (('pioglitazone'/exp OR 'pioglitazone':ab,ti OR '5 [4 [2 (5 ethyl 2 pyridyl) ethoxy] benzyl] 2, 4 thiazolidinedione':ab,ti OR '5 [ [4 [2 (5 ethyl 2pyridinyl) ethoxy] phenyl] methyl] 1, 3 thiazolidine 2, 4 dione':ab,ti OR '5 [ [4 [2 (5 ethylpyridin 2 yl) ethoxy] phenyl] methyl] 1, 3 thiazolidine 2, 4 dione':ab,ti OR 'actos':ab,ti OR 'ad 4833':ab,ti OR 'ad4833':ab,ti OR 'cereluc':ab,ti OR 'glidipion':ab,ti OR 'glita':ab,ti OR 'glitase':ab,ti OR

'glustin':ab,ti OR 'paglitaz':ab,ti OR 'pioglit':ab,ti OR 'pioglitazone hydrochloride':ab,ti OR 'pioglitazone potassium':ab,ti OR 'pioglitazone sodium':ab,ti OR 'pioglu':ab,ti OR 'piomed':ab,ti OR 'piozone':ab,ti OR 'sepioglin':ab,ti OR 'str001':ab,ti OR 'str 001 er':ab,ti OR 'str 001 it':ab,ti OR 'str001':ab,ti OR 'str001er':ab,ti

OR 'str001it':ab,ti OR 'u 72107':ab,ti OR 'u 72107a':ab,ti OR 'u 72107e':ab,ti OR 'u72107':ab,ti OR 'u72107a':ab,ti OR 'u72107e':ab,ti OR 'zactos':ab,ti) OR

(('treatment':ab,ti OR 'therapy':ab,ti) OR ((('sodium glucose cotransporter 2 inhibitor'/exp OR 'sodium glucose cotransporter 2 inhibitor':ab,ti OR 'gliflozin':ab,ti OR

'gliflozin derivative':ab,ti OR 'sglt2 inhibitors':ab,ti OR 'sodium-glucose transporter 2 inhibitors':ab,ti) OR ('canagliflozin'/exp OR 'canagliflozin':ab,ti OR '1, 5 anhydro 1 c [3 [5 (4 fluorophenyl) 2 thenyl] 4 methylphenyl] glucitol':ab,ti OR '1, 5 anhydro 1 c [3 [ [5 (4 fluorophenyl) 2 thienyl] methyl] 4 methylphenyl] d glucitol':ab,ti OR '1, 5 anhydro 1 c [3 [ [5 (4 fluorophenyl) thiophen 2 yl] methyl] 4 methylphenyl] d glucitol':ab,ti OR '2 [3 [5 (4 fluorophenyl) thiophen 2 ylmethyl] 4 methylphenyl] 6 (hydroxymethyl) tetrahydropyran 3, 4, 5 triol':ab,ti OR 'canagliflocin':ab,ti OR 'canagliflozin hemihydrate':ab,ti OR 'invokana':ab,ti OR 'jnj 28431754':ab,ti OR'jnj28431754':ab,ti OR 'ta 7284':ab,ti OR 'ta7284':ab,ti) OR ('dapagliflozin'/exp OR 'dapagliflozin':ab,ti OR '1 [4 chloro 3 (4 ethoxybenzyl) phenyl] 1 deoxy beta d

glucopyranose':ab,ti OR '2 (3 (4 ethoxybenzyl) 4 chlorophenyl) 6 hydroxymethyltetrahydro 2h pyran 3, 4, 5 triol':ab,ti OR '2 [4 chloro 3 (4 ethoxybenzyl) phenyl] 6 (hydroxymethyl) oxane 3, 4, 5 triol':ab,ti OR '2 [4 chloro 3 [ (4 ethoxyphenyl) methyl] phenyl] 6 (hydroxymethyl) oxane 3, 4, 5 triol':ab,ti OR 'bms 512148':ab,ti OR 'bms512148':ab,ti OR 'dapagliflozin acetate':ab,ti OR 'dapagliflozin propanediol':ab,ti OR 'dapagliflozin propanediol monohydrate':ab,ti OR 'edistride':ab,ti OR

'farxiga':ab,ti OR 'forxiga':ab,ti) OR ('ertugliflozin'/exp OR 'ertugliflozin':ab,ti OR '1, 6 anhydro 1 [4 chloro 3 (4 ethoxybenzyl) phenyl] 5 hydroxymethyl beta l idopyranose':ab,ti OR '1, 6 anhydro 1 c [4 chloro 3 [ (4 ethoxyphenyl) methyl] phenyl] 5 c (hydroxymethyl) beta l idopyranose':ab,ti OR '5 [4 chloro 3 (4 ethoxybenzyl) phenyl] 1 hydroxymethyl 6, 8 dioxabicyclo [3.2.1] octane 2, 3, 4 triol':ab,ti OR '5 [4 chloro 3 [ (4 ethoxyphenyl) methyl] phenyl] 1 (hydroxymethyl) 6, 8 dioxabicyclo

[3.2.1] octane 2, 3, 4 triol':ab,ti OR 'ertugliflozin pidolate':ab,ti OR 'ertugliflozin pyroglutamic acid':ab,ti OR 'mk 8835':ab,ti OR 'mk 8835':ab,ti OR 'pf 04971729':ab,ti OR 'pf 04971729 00':ab,ti OR 'pf 04971729-00':ab,ti OR 'pf 4971729':ab,ti OR 'pf 4971729 00':ab,ti OR 'pf 4971729-00':ab,ti OR 'pf04971729':ab,ti OR

'pf04971729 00':ab,ti OR 'pf04971729-00':ab,ti OR 'pf4971729':ab,ti OR 'pf4971729 00':ab,ti OR 'pf4971729-00':ab,ti OR 'steglatro':ab,ti) OR ('ipragliflozin'/exp OR 'ipragliflozin':ab,ti OR '1, 5 anhydro 1 c [3 [ (1 benzothiophen 2 yl) methyl] 4 fluorophenyl] dextro glucitol':ab,ti OR asp 1941':ab,ti OR 'asp1941':ab,ti) OR('luseogliflozin'/exp OR 'luseogliflozin':ab,ti OR '1, 5 anhydro 1 [5 (4 ethoxybenzyl) 2 methoxy 4 methylphenyl] 1 thio d glucitol':ab,ti OR '1, 5 anhydro 1 [5 (4 ethoxybenzyl) 2 methoxy 4 methylphenyl] 1 thio dextro glucitol':ab,ti OR '1, 5 anhydro 1 [5 (4 ethoxybenzyl) 2 methoxy 4 methylphenyl] 1 thioglucitol':ab,ti OR '2 [5 (4 ethoxybenzyl) 2 methoxy 4 methylphenyl] 6 (hydroxymethyl) tetrahydrothiopyran 3, 4, 5 triol':ab,ti OR '2 [5 [ (4 ethoxyphenyl) methyl] 2 methoxy 4 methylphenyl] 6 (hydroxymethyl) thiane 3, 4, 5 triol':ab,ti OR 'lusefi':ab,ti OR 'ts 071':ab,ti OR 'ts071':ab,ti) OR ('remogliflozin etabonate'/exp OR 'remogliflozin etabonate':ab,ti OR '4 (4 isopropoxybenzyl) 1isopropyl 5 methyl 1h pyrazol 3 yl 6 o (ethoxycarbonyl) beta dextro glucopyranoside':ab,ti OR '5 methyl 4 [4 (1 methylethoxy) benzyl] 1 (1 methylethyl) 1h pyrazol 3 yl 6 o (ethoxycarbonyl) beta dextro glucopyranoside':ab,ti OR 'gsk 189075':ab,ti OR 'gsk 189075a':ab,ti OR 'gsk189075':ab,ti OR 'gsk189075a':ab,ti) OR ('sotagliflozin'/exp OR 'sotagliflozin':ab,ti OR '2 [4 chloro 3 (4 ethoxybenzyl) phenyl] 6 (methylthio) tetrahydro 2h pyran 3, 4, 5 triol':ab,ti OR 'lp 802034':ab,ti OR 'lp802034':ab,ti OR 'lx 4211':ab,ti OR 'lx4211':ab,ti OR 'methyl 5 [4 chloro 3 (4 ethoxybenzyl) phenyl] 1 thio beta levo xylopyranoside':ab,ti OR 'methyl 5 [4 chloro 3 [ (4 ethoxyphenyl) methyl] phenyl] 1 thio beta levo xylopyranoside':ab,ti OR 'sar 439954':ab,ti OR 'sar439954':ab,ti OR 'zynquista':ab,ti) OR

('sergliflozin etabonate'/exp OR 'sergliflozin etabonate':ab,ti OR '2 (4 methoxybenzyl) phenyl 6 o (ethoxycarbonyl) beta dextro glucopyranoside':ab,ti OR '2 [ (4 methoxyphenyl) methyl] phenyl 6 o (ethoxycarbonyl) beta dextro glucopyranoside':ab,ti OR 'gw 869682':ab,ti OR 'gw 869682x':ab,ti OR 'gw869682':ab,ti OR 'gw869682x':ab,ti OR 'kgt 1251':ab,ti OR 'kgt1251':ab,ti OR 'sergliflozin':ab,ti) OR ('tofogliflozin'/exp OR 'tofogliflozin':ab,ti OR 'csg452':ab,ti OR 'rg 7201':ab,ti) OR

('empagliflozin'/exp OR 'empagliflozin':ab,ti OR '1, 5 anhydro 1 [4 chloro 3 [4 [ (tetrahydro 3 furyl) oxy] benzyl] phenyl] glucitol':ab,ti OR '1, 5 anhydro 1 [4 chloro 3 [ [4 [ (3 oxolanyl) oxy] phenyl] methyl] phenyl] glucitol':ab,ti OR '1, 5 anhydro 1 [4 chloro 3 [ [4 [ (tetrahydro 3 furanyl) oxy] phenyl] methyl] phenyl] glucitol':ab,ti OR '1, 5 anhydro 1 c [4 chloro 3 [ [4 [ (oxolan 3 yl) oxy] phenyl] methyl] phenyl] dextro glucitol':ab,ti OR '1, 5 anhydro 1 c [4 chloro 3 [ [4 [ (tetrahydro 3 furanyl) oxy] phenyl] methyl] phenyl] dextro glucitol':ab,ti OR 'bi 10773':ab,ti OR 'bi10773':ab,ti OR 'jardiance':ab,ti)) OR (('glucagon like peptide 1 receptor agonist'/exp OR 'glucagon like peptide 1 receptor agonist':ab,ti OR 'glp 1 agonist':ab,ti OR 'glp 1 receptor agonist':ab,ti OR 'glucagon like peptide 1 agonist':ab,ti OR 'glucagon like peptide 1 receptor stimulating agent':ab,ti OR 'long acting glp 1 agonist':ab,ti OR 'long acting glp 1 receptor agonist':ab,ti OR 'long acting glucagon like peptide 1 agonist':ab,ti OR 'long acting glucagon like peptide 1 receptor agonist':ab,ti) OR ('exendin 4'/exp OR 'exendin 4':ab,ti OR 'ac 2993':ab,ti OR 'ac 2993a':ab,ti OR 'ac2993':ab,ti OR 'ac2993a':ab,ti OR 'bydureon':ab,ti OR 'bydureon bcise':ab,ti OR 'bydureon pen':ab,ti OR 'byetta':ab,ti OR 'exenatide':ab,ti OR 'exenatide synthetic':ab,ti OR 'ly 2148568':ab,ti OR 'ly2148568':ab,ti) OR ('liraglutide'/exp OR 'liraglutide':ab,ti OR'glucagon like peptide 1 [7-37] [26 (6 n hexadecanoyl gamma glutamyllysine) 34 arginine]':ab,ti OR 'liraglutide recombinant':ab,ti OR 'n26 (hexadecanoyl gamma glutamyl) glucagon like peptide 1 [7-37] [34 arginine]':ab,ti OR 'nn 2211':ab,ti OR 'nn2211':ab,ti OR 'nnc 90 1170':ab,ti OR 'nnc 90-1170':ab,ti OR 'nnc90 1170':ab,ti OR 'nnc90-1170':ab,ti OR 'saxenda':ab,ti OR 'victoza':ab,ti) OR ('lixisenatide'/exp OR 'lixisenatide':ab,ti OR 'adlyxin':ab,ti OR 'aqve 10010':ab,ti OR 'aqve10010':ab,ti OR 'ave 0010':ab,ti OR 'ave0010':ab,ti OR 'des 38 proline

exendine 4 [1-39] peptidylpentalysyllysinamide':ab,ti OR 'lyxumia':ab,ti OR 'zp 10':ab,ti OR 'zp10':ab,ti) OR ('albiglutide'/exp OR 'albiglutide':ab,ti OR 'albugon':ab,ti OR 'albumin glp 1':ab,ti OR 'albumin glucagon like peptide 1':ab,ti OR 'albumin glucagon like peptide 1 fusion protein':ab,ti OR 'eperzan':ab,ti OR 'glp 1albumin':ab,ti OR 'glucagon like peptide 1 albumin':ab,ti OR 'glucagon like peptide 1 albumin fusion protein':ab,ti OR 'gsk 716155':ab,ti OR 'gsk 716155a':ab,ti OR'gsk-716155':ab,ti OR 'gsk-716155a':ab,ti OR 'gsk716155':ab,ti OR 'gsk716155a':ab,ti OR 'naliglutide':ab,ti OR 'syncria':ab,ti OR 'tanzeum':ab,ti) OR ('dulaglutide'/exp OR

'dulaglutide':ab,ti OR 'ly 2189265':ab,ti OR 'ly2189265':ab,ti OR 'trulicity':ab,ti) OR('semaglutide'/exp OR 'semaglutide':ab,ti OR'glucagon like peptide 1 [7-37] [8 (2 amino 2 methylpropanoic acid) 26 [6 n [18 [n (17 carboxyheptadecanoyl) gamma glutamyl] 10 oxo 3, 6, 12, 15 tetraoxa 9, 18 diazaoctadecanoyl] lysine] 34 arginine]':ab,ti OR 'nn 9535':ab,ti OR 'nn9535':ab,ti OR 'ozempic':ab,ti OR 'rybelsus':ab,ti)))) OR ('alpha tocopherol'/exp OR 'alpha tocopherol':ab,ti OR '5, 7, 8 trimethyltocol':ab,ti OR 'alpha tocopherol acetate':ab,ti OR 'alpha tocopherol glycolate':ab,ti OR 'alpha tocopherol palmitate':ab,ti OR 'alpha

tocopherolphosphate':ab,ti OR 'alpha tocopheryl acetate':ab,ti OR 'alpha tocopheryl acetic acid':ab,ti OR 'alpha-tocopherol':ab,ti OR 'alpha-tocopherol acetate':ab,ti OR 'aquasol e':ab,ti OR 'austrovit e':ab,ti OR 'covitol':ab,ti OR 'covitol 1360':ab,ti OR 'covitol 400c':ab,ti OR 'covitol f1000':ab,ti OR 'd 2, 5, 7, 8 tetramethyl 2 (4, 8, 12 trimethyltridecyl) chroman 6 ylacetate':ab,ti OR'd a tocopherol':ab,ti OR 'd alpha tocopherol':ab,ti OR 'd alpha tocopherol succinate':ab,ti OR 'd alphatocopherylacetate':ab,ti OR 'd vitamin e':ab,ti OR 'dagravit e':ab,ti OR 'dalfatol':ab,ti OR 'davitamon e':ab,ti OR 'dermorelle':ab,ti OR 'detulin':ab,ti OR 'dextro alpha tocopherol':ab,ti OR 'dextro vitamin e':ab,ti OR 'dextro, levo alpha tocopherol':ab,ti OR 'dl 2, 5, 7, 8 tetramethyl 2 (4, 8, 12 trimethyltridecyl) chroman 6 ylacetate':ab,ti OR 'dl alpha tocopherol':ab,ti OR 'dumovit e':ab,ti OR 'e ferol':ab,ti OR 'e perle':ab,ti OR 'e perte':ab,ti OR 'e recordati':ab,ti OR 'e toplex':ab,ti OR 'e vicotrat':ab,ti OR 'e vimin':ab,ti OR 'e vita':ab,ti OR 'e viterbin':ab,ti OR 'ecoferol':ab,ti OR 'efer':ab,ti OR 'eferol':ab,ti OR 'enoulan forte':ab,ti OR 'eplonat':ab,ti OR 'eprolin':ab,ti OR 'epsilan m':ab,ti OR 'epsylan m':ab,ti OR 'erevit':ab,ti OR 'erevit spofa':ab,ti OR 'esol; esorb':ab,ti OR 'eterapion':ab,ti OR 'eviabit':ab,ti OR 'evigen':ab,ti OR 'eviol':ab,ti OR 'evion':ab,ti OR 'evit':ab,ti OR 'evitol':ab,ti OR 'godabion e':ab,ti OR 'gonavit':ab,ti OR 'hanobak':ab,ti OR 'ido e':ab,ti OR 'juvela':ab,ti OR 'juvele':ab,ti OR 'levo alpha tocopherol':ab,ti OR 'livingpherol':ab,ti OR 'mixed tocopherols concentrate':ab,ti OR 'mulsal e':ab,ti OR 'natopherol':ab,ti OR 'optovit e':ab,ti OR 'phytoferol':ab,ti OR 'pletocol':ab,ti OR 'socopherol':ab,ti OR 'spondyvit':ab,ti OR 'toco 500':ab,ti OR 'tocoferolo bioglan':ab,ti OR 'tocomine':ab,ti OR 'tocopherex':ab,ti OR 'tocopherol acetate':ab,ti OR 'tocopherol gumpro':ab,ti OR 'tocopheryl acetate':ab,ti OR 'tocophrine':ab,ti OR 'tocovigor; tocovital':ab,ti OR 'toferol':ab,ti OR 'vi dom e':ab,ti OR 'vi e caps':ab,ti OR 'vi ea; vi etal':ab,ti OR 'vibolex e':ab,ti OR 'vidom e':ab,ti OR 'viea; vietal':ab,ti OR 'viprimol':ab,ti OR 'vita e gelucaps':ab,ti OR 'vitamin e':ab,ti OR 'vitamin e acetate':ab,ti OR 'vitamin e palmitate':ab,ti OR 'viteolin':ab,ti OR 'viteoline':ab,ti OR 'wandervit e':ab,ti))

**Cochrane Library**

#1 MeSH descriptor: [Sodium-Glucose Transporter 2 Inhibitors] explode all trees

#2 (Sodium-Glucose Transporter 2 Inhibitor):ti,ab,kw OR (Inhibitor, SGLT-2):ti,ab,kw OR (SGLT 2 Inhibitor SGLT 2 Inhibitors):ti,ab,kw OR (SGLT-2 Inhibitor):ti,ab,kw OR (Sodium Glucose Transporter 2 Inhibitor):ti,ab,kw

#3 (Gliflozin):ti,ab,kw OR (SGLT-2 Inhibitors):ti,ab,kw OR (Sodium Glucose Transporter 2 Inhibitors):ti,ab,kw OR (Gliflozins):ti,ab,kw OR (SGLT2 Inhibitor):ti,ab,kw

#4 (Inhibitor, SGLT2):ti,ab,kw OR (SGLT2 Inhibitors):ti,ab,kw

#5 #1 or #2 or #3 or #4

#6 MeSH descriptor: [Canagliflozin] explode all trees

#7 (Canagliflozin, Anhydrous):ti,ab,kw OR (Canagliflozin Hemihydrate):ti,ab,kw OR (Invokana):ti,ab,kw OR (Canagliflozin):ti,ab,kw

#8 #6 or #7

#9 #5 or #8

#10 MeSH descriptor: [Glucagon-Like Peptide-1 Receptor] explode all trees

#11 (Glucagon Like Peptide 1 Receptor):ti,ab,kw OR (Peptide-1 Receptor, Glucagon-Like):ti,ab,kw OR (Receptor, Glucagon-Like Peptide-1):ti,ab,kw OR (Receptor, GLP-1):ti,ab,kw OR (GLP-1R Receptor):ti,ab,kw

#12 (GLP-1 Receptor):ti,ab,kw OR (GLP 1 Receptor):ti,ab,kw OR (Receptor, GLP-1R):ti,ab,kw OR (GLP 1R Receptor):ti,ab,kw OR (GLP1R Receptor):ti,ab,kw

#13 (GLP1R Protein):ti,ab,kw OR (Receptor, GLP1R):ti,ab,kw OR (Protein, GLP1R):ti,ab,kw OR (Glucagon-Like Peptide-1 Receptor):ti,ab,kw

#14 #10 or #11 or #12 or #13

#15 #10 or #11 or #12 or #13

#16 (Peptide, Ex4):ti,ab,kw OR (Exendin-4):ti,ab,kw OR (Ex4 Peptide):ti,ab,kw OR (ITCA 650):ti,ab,kw OR (AC 2993 LAR):ti,ab,kw

#17 (Exendin 4):ti,ab,kw OR (AC 2993):ti,ab,kw OR (Byetta):ti,ab,kw OR (Bydureon):ti,ab,kw OR (Exenatide):ti,ab,kw

#18 #15 or #16 or #17

#19 MeSH descriptor: [Liraglutide] explode all trees

#20 (Victoza):ti,ab,kw OR (Saxenda):ti,ab,kw OR (NN-2211):ti,ab,kw OR (NN 2211):ti,ab,kw OR (NN2211):ti,ab,kw

#21 (2211, NN):ti,ab,kw OR (Liraglutide):ti,ab,kw

#22 #19 or #20 or #21

#23 #14 or #18 or #22

#24 MeSH descriptor: [Pioglitazone] explode all trees

#25 (U72,107A):ti,ab,kw OR (U72107A):ti,ab,kw OR (U-72107A):ti,ab,kw OR (U 72107A):ti,ab,kw OR (AD 4833):ti,ab,kw

#26 (AD-4833):ti,ab,kw OR (AD4833):ti,ab,kw OR (Pioglitazone Hydrochloride):ti,ab,kw OR (Actos):ti,ab,kw OR (Pioglitazone):ti,ab,kw

#27 #24 or #25 or #26

#28 MeSH descriptor: [Vitamin E] explode all trees

#29 MeSH descriptor: [Tocopherols] explode all trees

#30 MeSH descriptor: [alpha-Tocopherol] explode all trees

#31 (Vitamin E):ti,ab,kw

#32 #28 or #31

#33 (Vitamin E Suspension):ti,ab,kw OR (Vitamin E Natur):ti,ab,kw OR (Togasan Vitamin E):ti,ab,kw OR (Vitamin E, Togasan):ti,ab,kw OR (Mowivit Vitamin E):ti,ab,kw

#34 (Vitamin E, Mowivit):ti,ab,kw OR (Vitagutt Vitamin E):ti,ab,kw OR (Vitamin E, Vitagutt):ti,ab,kw OR (EUNOVA Vitamin E):ti,ab,kw OR (Vitamin E Sanum):ti,ab,kw

#35 (Vitamin-E Dragees):ti,ab,kw OR (Vitamin E Dragees):ti,ab,kw OR (Vitamin E-mp):ti,ab,kw OR (Vitamin E mp):ti,ab,kw OR (E Vitamin E):ti,ab,kw

#36 (Vitamin-E EVI-MIRALE):ti,ab,kw OR (Vitamin E EVI MIRALE):ti,ab,kw OR (Vitamin E AL):ti,ab,kw OR (Vitamine E GNR):ti,ab,kw OR (Tocopherols):ti,ab,kw

#37 #29 or #33 or #34 or #35 or #36

#38 (alpha-Tocopherol):ti,ab,kw OR (Vitamin E Succinate):ti,ab,kw

#39 #30 or #38

#40 #32 or #37 or #39

#41 MeSH descriptor: [Non-alcoholic Fatty Liver Disease] explode all trees

#42 (Liver, Nonalcoholic Fatty):ti,ab,kw OR (NAFLD):ti,ab,kw OR (Non alcoholic Fatty Liver Disease):ti,ab,kw OR (Nonalcoholic Fatty Liver Disease):ti,ab,kw OR (Fatty Liver, Nonalcoholic):ti,ab,kw

#43 (Nonalcoholic Fatty Liver):ti,ab,kw OR (Nonalcoholic Fatty Livers):ti,ab,kw OR (Livers, Nonalcoholic Fatty):ti,ab,kw OR (Fatty Livers, Nonalcoholic):ti,ab,kw OR (Steatohepatitides, Nonalcoholic):ti,ab,kw

#44 (Nonalcoholic Steatohepatitis):ti,ab,kw OR (Steatohepatitis, Nonalcoholic):ti,ab,kw OR (Nonalcoholic Steatohepatitides):ti,ab,kw OR (Non-alcoholic Fatty Liver Disease):ti,ab,kw

#45 #41 or #42 or #43 or #44

#46 (compare):ti,ab,kw OR (compared):ti,ab,kw OR (comparison):ti,ab,kw OR (comparative):ti,ab,kw OR (comparing):ti,ab,kw

#47 (versus):ti,ab,kw OR (vs):ti,ab,kw

#48 #46 or #47

#49 (Randomized Controlled Trial):pt OR (controlled clinical trial):pt

#50 (randomized):ti,ab,kw OR (randomised):ti,ab,kw OR (randomly):ti,ab,kw OR (trial):ti,ab,kw OR (phase):ti,ab,kw

#51 #49 or #50

#52 (treatment):ti,ab,kw OR (therapy):ti,ab,kw

#53 #9 or #23 or #27 or #40 or #52

#54 #45 and #48 and #51

#55 #53 and #54

**Web of Science**

#11

#10 AND #4

#10

#9 OR #8 OR #7 OR #6 OR #5

#9

TS=(treatment OR therapy)

#8

TS=(Gonavit OR godabion e OR Evitol OR Evit OR Evion OR Eviol OR Evigen OR Eviabit OR eterapion OR esol; esorb OR erevit spofa OR erevit OR epsylan m OR epsilan OR Eprolin OR EplonatOR enoulan forte OR eferol OR efer OR ecoferol OR e viterbin OR e vita OR e vimin OR e vicotrat OR e toplex OR e recordati OR e perte OR e perle OR e ferol OR dumovit e OR dl alpha tocopherol OR dl 2, 5, 7, 8 tetramethyl 2 (4, 8, 12 trimethyltridecyl) chroman 6 ylacetate OR dextro, levo alpha tocopherol OR dextro vitamin e OR dextro alpha tocopherol OR dermorelle OR davitamon e OR dalfatol OR dagravit e OR d vitamin e OR d alphatocopherylacetate OR d alpha tocopherol succinate OR d a tocopherol':ab,ti OR 'd alpha tocopherol OR d 2, 5, 7, 8 tetramethyl 2 (4, 8, 12 trimethyltridecyl) chroman 6 ylacetate OR covitol f1000 OR covitol 400c OR covitol 1360 OR covitol OR austrovit e OR aquasol e OR alpha-tocopherol acetate OR alpha-tocopherol OR alpha tocopheryl acetate OR alpha tocopherolphosphate OR alpha tocopherol palmitate OR alpha tocopherol glycolate OR alpha tocopherol acetate OR trimethyltocol OR alpha tocopherolOR viea; vietal OR vidom e OR vibolex e OR vi ea; vi etal OR vi e caps OR Toferol OR vi dom e OR tocovigor; tocovital OR Tocophrine OR tocopheryl acetate OR tocopherol gumpro OR tocopherol acetate OR Tocopherex OR tocomine OR tocoferolo bioglan OR toco 500 OR Spondyvit OR Socopherol OR pletocol OR phytoferol OR optovit e OR Natopherol OR mulsal e OR mixed tocopherols concentrate OR Livingpherol OR levo alpha tocopherol OR Juvele OR Juvela OR ido e OR hanobak OR wandervit e OR Viteoline OR vitamin e acetate OR vitamin e palmitate OR Viteolin OR vitamin e OR vita e gelucaps OR Viprimol )

#7

| TS=(5-(4-(2-(5-Ethyl-2-pyridyl)ethoxy)benzyl)-2,4-thiazolidinedione OR U 72107A OR U72,107A OR U-72107A OR U72107A OR AD 4833 OR AD-4833 OR AD4833 OR Pioglitazone Hydrochloride OR Actos OR Pioglitazone) |
| --- |

#6

TS=(Glucagon Like Peptide 1 Receptor OR GLP-1R Receptor OR GLP 1R Receptor OR GLP1R Protein OR GLP-1 Receptor OR GLP 1 Receptor OR GLP1R Receptor OR Glucagon-Like Peptide-1 Receptor OR Bydureon OR ITCA 650 OR AC 2993 LAR OR Exendin-4 OR Ex4 Peptide OR Exendin 4 OR Byetta OR AC 2993 OR Exenatide OR Victoza OR Saxenda OR NN 2211 OR 2211, NN OR NN2211OR NN-2211 OR Liraglutide OR DES-38-proline-exendine-4 (Heloderma suspectum) -(1-39)-peptidylpenta-l-lysyl-l-lysinamide OR Adlyxin OR AQVE-10010 OR ZP10A peptide OR ZP 10 OR ZP-10 OR Lyxumia OR AVE 010 OR AVE-010 OR AVE 0010 OR AVE0010 OR AVE-0010 OR lixisenatide OR Eperzan OR Tanzeum OR albiglutide OR rGLP-1 protein OR LY 2189265 OR LY-2189265 OR LY2189265 OR Trulicity OR dulaglutide OR rybelsus OR Ozempic OR semaglutide )

#5

| TS=(Sodium-Glucose Transporter 2 Inhibitors OR Sodium Glucose Transporter 2 OR Sodium-Glucose Transporter 2 Inhibitor OR Sodium Glucose Transporter 2 Inhibitor OR SGLT-2 Inhibitors OR SGLT 2 Inhibitors OR Gliflozins OR SGLT2 Inhibitors OR Gliflozin OR SGLT-2 Inhibitor OR SGLT 2 Inhibitor OR SGLT2 Inhibitor OR Invokana OR Canagliflozin Hemihydrate OR Canagliflozin, Anhydrous OR 1-(Glucopyranosyl)-4-methyl-3-(5-(4-fluorophenyl)-2-thienylmethyl)benzene - T777973[Title/Abstract] OR Sodium-Glucose Transporter 2 Inhibitors OR dapagliflozin OR (2S,3R,4R,5S,6R) -2-(4-chloro-3-(4-ethoxybenzyl)phenyl)-6- (hydroxymethyl) tetrahydro-2H-pyran-3,4,5-triol OR Farxiga[Title/Abstract] OR Forxiga OR Forxiga OR 2-(3-(4-ethoxybenzyl)-4-chlorophenyl)-6-hydroxymethyltetrahydro-2H-pyran-3,4,5-triol[Title/Abstract] OR BMS 512148 OR BMS512148 OR BMS-512148 OR 5-(4-chloro-3-(4-ethoxybenzyl)phenyl)-1-hydroxymethyl-6,8-dioxabicyclo(3.2.1)octane-2,3,4-triol OR Steglatro OR PF 04971729 OR PF04971729 OR PF-04971729 OR ertugliflozin OR (1S) -1,5-anhydro-1-(3-(1-benzothiophen-2-ylmethyl)-4-fluorophenyl)-D-glucitol OR Suglat OR ASP1941 OR ASP-1941 OR ipragliflozin OR 4-(4-isopropoxybenzyl)-1-isopropyl-5-methyl-1H-pyrazol-3-yl 6-O-(ethoxycarbonyl)-beta-D-glucopyranoside OR remogliflozin etabonate OR sotagliflozin OR LX4211 OR LX-4211 OR (2S,3R,4R,5S,6R) -2-(4-chloro-3-(4-ethoxybenzyl)phenyl)-6-(methylthio)tetrahydro-2H-pyran-3,4,5-triol OR sergliflozin OR tofogliflozin hydrate OR CSG452 OR tofogliflozin anhydrous OR Apleway OR Deberza OR tofogliflozin OR 6-((4-ethylphenyl)methyl)-3',4',5',6'-tetrahydro-6'-(hydroxymethyl)spiro(isobenzofuran-1(3H),2'-(2H)pyran)-3',4',5'-triol OR 1-chloro-4-(glucopyranos-1-yl)-2-(4-(tetrahydrofuran-3-yloxy)benzyl)benzene OR BI 10773 OR BI10773 OR BI-10773 OR Jardiance OR empagliflozin) |
| --- |

#4

#3 AND #2 AND #1

#3

TS=(Randomized Controlled Trial OR controlled clinical trial OR randomized OR randomised OR randomly OR trial OR phase)

#2

TS=(compare OR compared OR comparison OR comparative OR comparing OR versus OR vs)

#1 TS=(Non-alcoholic Fatty Liver Disease OR Non alcoholic Fatty Liver Disease OR NAFLD OR Nonalcoholic Fatty Liver Disease OR Nonalcoholic Fatty Liver OR Nonalcoholic Fatty Livers OR Nonalcoholic Steatohepatitis OR Nonalcoholic Steatohepatitides)
